# A Negative Feedback Modulator of Antigen Processing Evolved from a Frameshift in the Cowpox Virus Genome

**DOI:** 10.1371/journal.ppat.1004554

**Published:** 2014-12-11

**Authors:** Jiacheng Lin, Sabine Eggensperger, Susanne Hank, Agnes I. Wycisk, Ralph Wieneke, Peter U. Mayerhofer, Robert Tampé

**Affiliations:** 1 Institute of Biochemistry, Biocenter, Goethe-University Frankfurt, Frankfurt, Germany; 2 Cluster of Excellence – Macromolecular Complexes, Goethe-University Frankfurt, Frankfurt, Germany; Harvard Medical School, United States of America

## Abstract

Coevolution of viruses and their hosts represents a dynamic molecular battle between the immune system and viral factors that mediate immune evasion. After the abandonment of smallpox vaccination, cowpox virus infections are an emerging zoonotic health threat, especially for immunocompromised patients. Here we delineate the mechanistic basis of how cowpox viral CPXV012 interferes with MHC class I antigen processing. This type II membrane protein inhibits the coreTAP complex at the step after peptide binding and peptide-induced conformational change, in blocking ATP binding and hydrolysis. Distinct from other immune evasion mechanisms, TAP inhibition is mediated by a short ER-lumenal fragment of CPXV012, which results from a frameshift in the cowpox virus genome. Tethered to the ER membrane, this fragment mimics a high ER-lumenal peptide concentration, thus provoking a trans-inhibition of antigen translocation as supply for MHC I loading. These findings illuminate the evolution of viral immune modulators and the basis of a fine-balanced regulation of antigen processing.

## Author Summary

Virus-infected or malignant transformed cells are eliminated by cytotoxic T lymphocytes, which recognize antigenic peptide epitopes in complex with major histocompatibility complex class I (MHC I) molecules at the cell surface. The majority of such peptides are derived from proteasomal degradation in the cytosol and are then translocated into the ER lumen in an energy-consuming reaction *via* the transporter associated with antigen processing (TAP), which delivers the peptides onto MHC I molecules as final acceptors. Viruses have evolved sophisticated strategies to escape this immune surveillance. Here we show that the cowpox viral protein CPXV012 inhibits the ER peptide translocation machinery by allosterically blocking ATP binding and hydrolysis by TAP. The short ER resident active domain of the viral protein evolved from a reading frame shift in the cowpox virus genome and exploits the ER-lumenal negative feedback peptide sensor of TAP. This CPXV012-induced conformational arrest of TAP is signaled by a unique communication across the ER membrane to the cytosolic motor domains of the peptide pump. Furthermore, this study provides the rare opportunity to decipher on a molecular level how nature plays hide and seek with a pathogen and its host.

## Introduction

Coexistence of pathogens and their hosts represents a masterpiece of evolution, which relies on a fine-tuned balance between pathogen replication and clearance of pathogens by the host immune system [1]. To escape immune surveillance, viruses have developed sophisticated strategies [2], [3]. For example, Herpes simplex viruses and varicella-zoster virus establish latency in trigeminal and dorsal root ganglia, which express only low levels of major histocompatibility complex class I (MHC I) molecules [2], [4], [5]. Exploitation refers *e.g.* to memory T-cells that circulate through the body and hence provide excellent vehicles for virus dissemination during primary simian varicella virus infection [6]. Sabotage is mediated *e.g.* by a C-type lectin-like gene product of cytomegalovirus that functions as a decoy ligand to subvert missing-self recognition by natural killer cells (NK), thereby circumventing the elimination of the virus infected cell [5], [7].

Recognizing virus-specific epitopes displayed on MHC I at the cell surface is the essential step in priming and execution of an adaptive immune response against infection. These antigenic peptide epitopes are derived from degradation of the cellular proteome, including virus or tumor associated gene products, *via* the ubiquitin-proteasomal pathway. The generated peptides are translocated into the ER lumen by the transporter associated with antigen processing (TAP) and subsequently loaded onto MHC I molecules [5], [8]. This heterodimeric ATP-binding cassette (ABC) transport complex is composed of two transmembrane domains (TMDs) and two cytosolic nucleotide-binding domains (NBDs), which couple the chemical energy of ATP binding and hydrolysis to the peptide translocation across the ER membrane [9]. TAP is the central component of the peptide-loading complex (PLC), composed of TAP1/2, tapasin (Tsn), ERp57, calreticulin, and MHC I. After guiding antigenic peptides to MHC I molecules [5], [10], peptide-loaded MHC I complexes dissociate from the PLC and traffic *via* the secretory pathway to the cell surface, where their antigenic cargo is inspected by cytotoxic T lymphocytes (CTLs). TAP can be dissected into the coreTAP complex, which has been shown to be essential and sufficient for peptide translocation, and extra N-terminal transmembrane domains (TMD0), which bind Tsn and are essential for the assembly of the PLC [11]–[14].

CPXV012 encoded by cowpox viruses (CPXV) has been identified to inhibit antigen processing, extending the types of viruses beyond the *Herpesviridae* family that interfere with the delivery of antigenic peptides into the ER lumen [15], [16]. CPXV012 encodes an ER-resident type II membrane protein [15] of 69 amino acids, harboring a signal anchor sequence and a short C-terminal region in the ER lumen. CPXV012 associates with the PLC and prevents peptide loading onto MHC I molecules by inhibiting peptide translocation into the ER lumen [15], [16]. Notably, CPXV012 prevents CD8^+^ T-cell effector responses by inhibiting peptide translocation into the ER [15], [16].

CPXV belongs to *Orthopoxvirus* (OPV) genus of the *Poxviridae* family that also includes clinically relevant pathogens such as the variola virus, which causes smallpox. In contrast to herpesviruses, poxviruses are acute viruses that use a “kiss-and-run” strategy of propagation among host cells. CPXV can infect and replicate in the cells of many different mammalian species, including humans. In Europe and parts of Asia, endemic CPXV is the most common cause of human OPV infections [17]. The increased number of CPXV infections indicates that the abandonment of the smallpox vaccination in 1977 may render the population more vulnerable to CPXV. This emerging zoonotic hazard for humans raises public health concerns. CPXV infections of healthy humans are generally self-limiting and cause only localized skin lesions [18]. However, severe CPXV infections with lethal outcome have been reported in immune compromised and eczematous patients, particularly children [19], [20].

Given the medical relevance, it is of particular interest to understand the molecular basis of how CPXV mediates immune evasion. Here we identified the coreTAP complex as the direct target for CPXV012, and revealed how antigen translocation is mechanistically blocked. We further provide an explanation on how this poxviral factor has evolved its evasion potential to escape surveillance by the adaptive immune system.

## Results

### CPXV012 inhibits MHC I antigen presentation by directly targeting TAP

**Figure 1:**
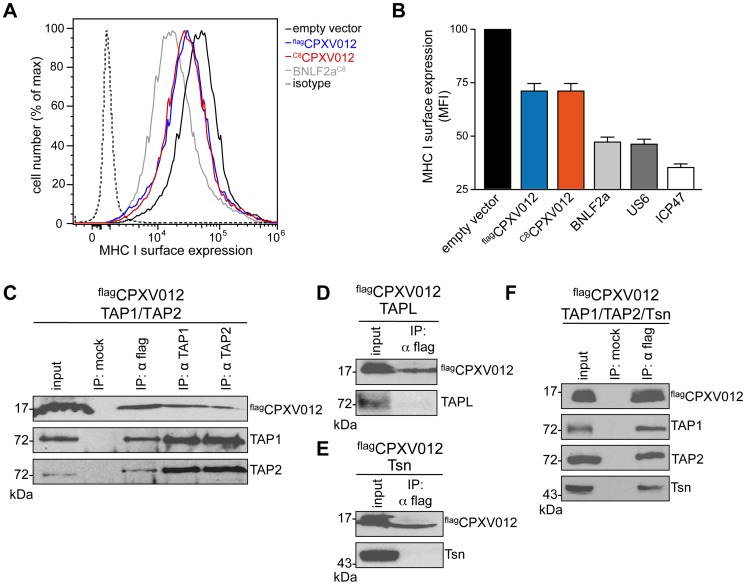
CPXV012 inhibits MHC I antigen presentation by targeting TAP directly. (A) Down-regulation of MHC I surface expression by CPXV012. HeLa cells were transiently transfected with empty vector, ^flag^CPXV012, ^C8^CPXV012, BNLF2a^C8^, US6^myc^ or ICP47 in pIRES2-EGFP, respectively. For clarity, US6 and ICP47 are summarized only in panel B. Peptide-loaded MHC I molecules at the cell surface were analyzed by flow cytometry. Only GFP-positive cells were analyzed; isotype control (dotted line). (B) Evaluation of the histograms from A. Mean fluorescence intensity (MFI) was calculated for cells transfected with the indicated constructs. (C–F) CPXV012 binds directly to TAP. ^flag^CPXV012 was coexpressed with TAP1/2 (C), TAPL (D), Tsn (E), or TAP1/2 and Tsn (F) in *Sf*9 cells. Proteins were immunoprecipitated with TAP1 (mAb 148.3), TAP2 (mAb 435.3), or flag-specific antibodies (IP). The HC10-antibody was used as negative control (mock). Samples were analyzed by immunoblotting with the corresponding antibodies. An aliquot (1/20) of the crude membrane input is shown.

We first examined whether CPXV012 variants affect the antigen presentation monitored by MHC I surface expression. As shown by flow cytometry, both Flag-tagged ^flag^CPXV012 and C8-tagged ^C8^CPXV012 caused a down-regulation of MHC I surface expression in HeLa cells (Fig. 1A and S1 Figure). The expression of other known immune evasins, such as ICP47 of herpes simplex virus type 1 (HSV-1) that binds to the cytosolic face of the TAP complex and blocks peptide binding [21]–[23], the human cytomegalovirus (HCMV) type I membrane glycoprotein US6 that inhibits ATP binding by TAP *via* its ER-lumenal domain [24], [25], and the tail-anchored Epstein-Barr virus (EBV) protein BNLF2a that prevents peptide and ATP binding to TAP [26]–[29] had an even stronger effect on MHC I processing (Fig. 1B and S1 Figure). Other than herpesviridae that are known to express highly potent TAP inhibitors, CPXV might rely on a synergistic cooperation between CPXV012 and CPXV203, which blocks MHC I trafficking [30], to efficiently down-regulate MHC I expression at the cell surface.

CPXV012 has been reported to associate with the murine PLC without disrupting its overall assembly [16]; however, the direct interaction partner within this macromolecular complex has not been identified so far. We therefore expressed ^flag^CPXV012 or ^C8^CPXV012 in insect cells, which provide the key advantage that any combination of PLC subunits can be expressed in a cellular background that lacks all components of the PLC [11]. As shown by coimmunoprecipitation using TAP1, TAP2, or flag specific antibodies, CPXV012 associated directly with the TAP complex (Fig. 1C). Similar results were obtained for C8-tagged ^C8^CPXV012 (see below). The interaction with TAP is specific, because CPXV012 did not coprecipitate with TAPL (Fig. 1D), a lysosomal peptide translocation complex, which shares approx. 40% amino acid sequence identity with TAP1 or TAP2 [31]. Recent studies proposed that CPXV012 interacts with Tsn, thereby impairing Tsn-mediated peptide binding to MHC I [15], [16]. Notably, Tsn was coprecipitated with CPXV012 only in the presence of the TAP complex. These results suggest that CPXV012 does not directly interact with Tsn (Fig. 1E), but is bound to the PLC *via* its direct interaction with TAP (Fig. 1F). Moreover, the presence of CPXV012 does not block the TAP-Tsn interaction.

### CPXV012 blocks antigen translocation by coreTAP after peptide binding

**Figure 2:**
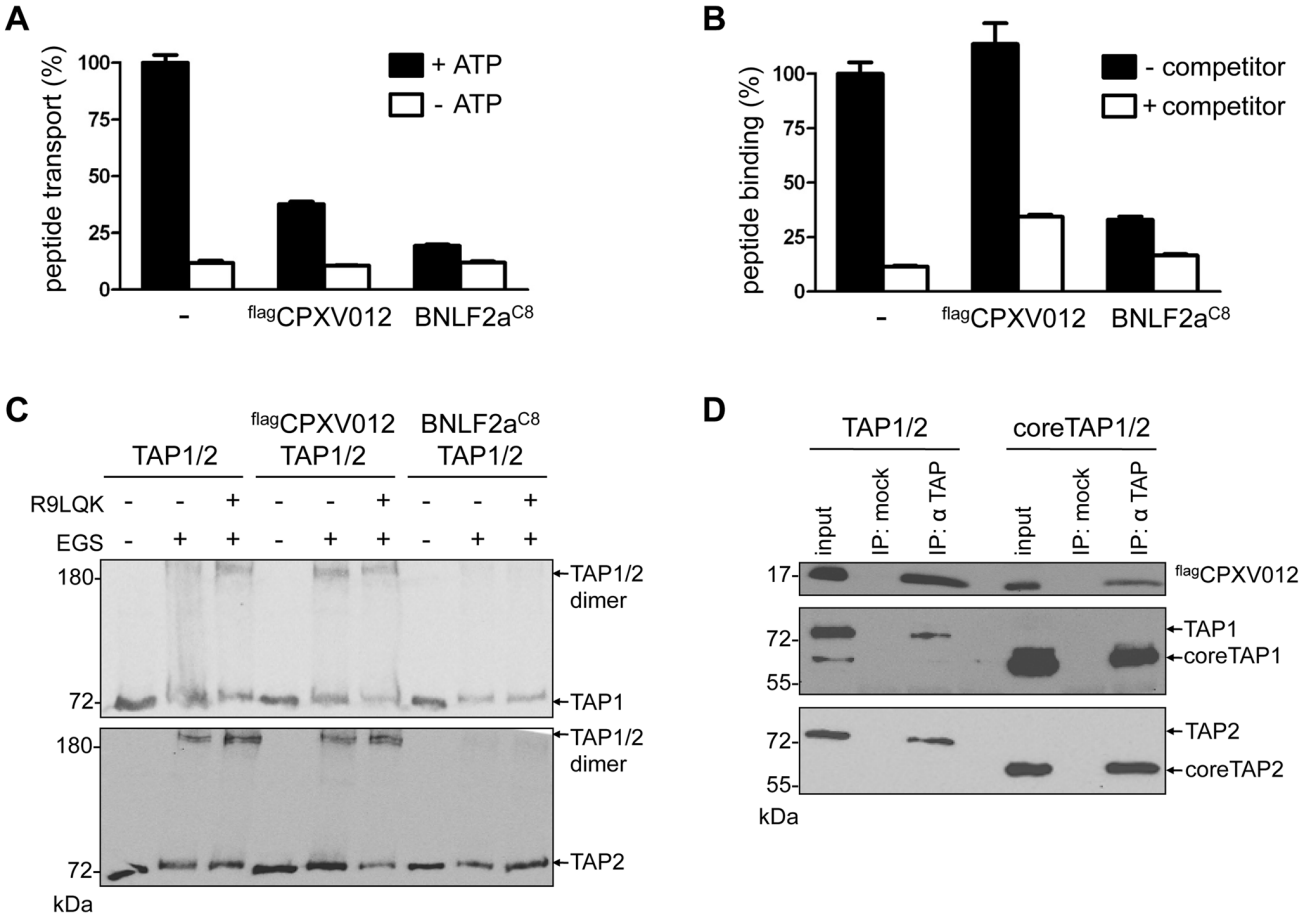
CPXV012 inhibits peptide transport but not peptide binding to TAP. TAP1, TAP2, ^flag^CPXV012, and BNLF2a^C8^ were coexpressed in *Sf*9 cells as indicated. (A) CPXV012 inhibits peptide transport of TAP. Crude membranes were incubated with RRYQNSTC^(F)^L peptide (C^(F)^, fluorescein-labeled cysteine; N-core glycosylation site underlined) in the absence or presence of ATP. Translocated and N-core glycosylated peptides were bound to ConA-beads and quantified by fluorescence. Peptide transport by TAP was normalized to 100%. The means of at least three independent experiments are shown. Error bars indicate the S.D. (B) CPXV012 does not inhibit peptide binding to TAP. Crude membranes were incubated with high-affinity peptide RRYC^(F)^KSTEL (filled bars). A 100-fold excess of RRYQKSTEL (R9LQK) was used to probe for unspecific binding (open bars). Bound peptides were quantified by fluorescence. The membranes used for (A) and (B) expressed similar amounts of TAP1/2 (S1 Figure). (C) CPXV012 does not interfere with a peptide-induced conformational change of TAP. Membranes were incubated in the presence or absence of the peptide R9LQK. After EGS cross-linking, samples were analyzed by SDS-PAGE (6%) and immunoblotting with TAP1 (mAb 148.3) and TAP2 (mAb 435.3) specific antibodies. (D) CPXV012 binds to coreTAP. ^flag^CPXV012 was coexpressed with either full-length TAP1/2 (left) or coreTAP1/2 (right). Proteins were immunoprecipitated with a mixture of polyclonal TAP1 (1p2) and TAP2 specific (2p4) antibodies (IP). The anti-US6 antibody was used a negative control (mock). Samples were analyzed by SDS-PAGE (12%) and subsequent immunoblotting with either anti-flag, monoclonal anti-TAP1 (mAb 148.3), or anti-TAP2 (mAb 435.3) antibodies. An aliquot (1/20) of the crude membrane input is shown.

CPXV012 inhibits the TAP-mediated peptide translocation into the ER lumen in the absence of other components of the PLC (Fig. 2A). As reported previously, a more pronounced inhibitory effect was observed for the herpesviral inhibitors UL49.5 [16] and EBV-BNLF2a. In contrast to BNLF2a, which blocks both, peptide binding and translocation [27]–[29], [32], CPXV012 did not interfere with peptide binding to TAP (Fig. 2B). Upon peptide binding, rearrangements within the TMDs of TAP induce a conformational change of the NBDs, which can be monitored by cross-linking using the amine-specific homobifunctional reagent EGS [24], [33]. Hence, we examined the influence of CPXV012 on the peptide-induced conformational change by EGS cross-linking. In the presence of peptides, cross-linking of the TAP1/2 heterodimer was detected at ∼160 kDa (Fig. 2C). In the presence of EBV-BNLF2a, the peptide-induced cross-linking of the TAP heterodimer is abolished [29]. In contrast, CPXV012 had no effect on the peptide-induced conformational change monitored by cross-linking of TAP1 and TAP2.

As the coreTAP complex is essential and sufficient for peptide binding and translocation [11], we also examined whether CPXV012 can block this minimal functional unit of antigen translocation. Interestingly, CPXV012 inhibited peptide translocation *via* coreTAP in a similar way as the full TAP complex (S2A Figure). Consistent with the data obtained for full-length TAP, CPXV012 did not interfere with peptide binding to coreTAP (S2B Figure). As demonstrated by coimmunoprecipitation using TAP1 and TAP2 specific antibodies, CPXV012 binds specifically to coreTAP (Fig. 2D). Taken together, our data demonstrate that CPXV012 interferes with antigen processing by blocking the coreTAP complex at a state after peptide binding and a peptide-induced conformational change.

### CPXV012 inhibits ATP binding by TAP1 and TAP2

**Figure 3:**
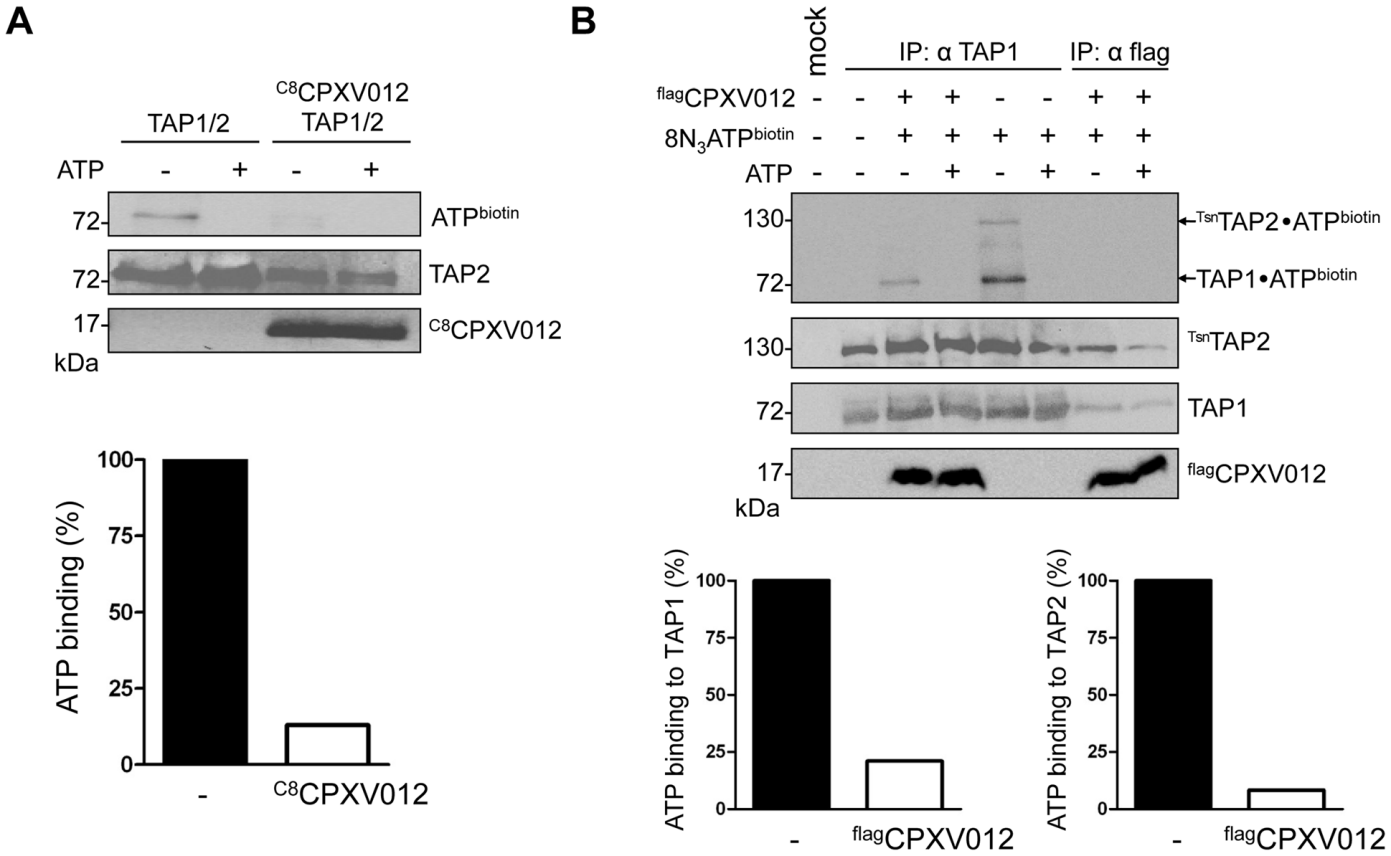
CPXV012 blocks ATP binding to TAP1 and TAP2. (A) CPXV012 inhibits ATP binding to TAP. TAP1, TAP2, and ^C8^CPXV012 were coexpressed in *Sf*9 cells as indicated. TAP1/TAP2 or TAP1/TAP2/^C8^CPXV012 complexes were affinity purified using anti-TAP1 (mAb 148.3) or anti-C8 antibodies, respectively. Dynabead immobilized proteins were pre-incubated with 15 µM 8-azido-ATP[γ]biotin in the presence or absence of 5 mM ATP for 5 min on ice and subsequently subjected to UV irradiation. Dynabead immobilized proteins were then separated by SDS-PAGE and detected by immunoblotting with the antibodies as indicated. Biotinylated proteins were visualized using extravidin-HRP conjugate. In the presence (open bars) and absence of ^C8^CPXV012 (filled bars), the amount of ATP cross-linked TAP was normalized to the TAP2 protein expression levels. (B) CPXV012 inhibits ATP binding sites to TAP1 and TAP2. TAP1, ^Tsn^TAP2, and ^flag^CPXV012 were coexpressed and crude membranes were incubated with 8-azido-ATP[γ]biotin (15 µM) in the presence or absence of ATP (5 mM) for 5 min on ice. After UV irradiation, proteins were immunoprecipitated with TAP1 (mAb 148.3) or flag-specific antibodies (IP). The HC10-antibody was used as negative control (mock). Immunoprecipitated samples were then separated by SDS-PAGE and analyzed by immunoblotting with extravidin-HRP or the corresponding antibodies. The amount of TAP photo cross-linked by 8-azido-ATP in the absence (filled bars) or presence (open bars) of ^flag^CPXV012 was normalized to TAP1 or ^Tsn^TAP2 protein expression levels, respectively.

To shed further light on the inhibition mechanism of CPXV012, we examined whether the TAP1/2•CPXV012 complex can bind ATP. TAP1/2 or TAP1/2•^C8^CPXV012 complexes were affinity purified and subsequently photo cross-linked with 8-azido-ATP[γ]biotin. Cross-linked proteins were analyzed by immunoblotting using anti-biotin extravidin-HRP. A single biotinylated band at 70-kDa was observed in the absence of ^C8^CPXV012, consistent with azido-ATP photo labeling of TAP1 or TAP2 (Fig. 3A). The photo labeling of TAP was specific, because it was blocked by an access of unlabeled ATP. Strikingly, ATP cross-linking was inhibited at the TAP1/2•CPXV012 complex. These results demonstrate that CPXV012 inhibits ATP binding or the rate of nucleotide exchange by TAP.

Notably, the TAP complex harbors a canonical ATP-binding site II and a non-canonical ATP-binding site I. However, the functional role of these non-equivalent sites is not completely understood [9]. Hence, we investigated whether CPXV012 affects ATP binding to both sites. To distinguish between TAP1 (71 kDa) and TAP2 (72 kDa) in SDS-PAGE, the N-terminus of TAP2 was fused to tapasin (Tsn) *via* a flexible linker of 34 amino acids (^Tsn^TAP2). It is worth mentioning that the TAP1/^Tsn^TAP2 transport complex is fully functional in peptide translocation and MHC I peptide loading [14]. Moreover, expression of ^flag^CPXV012 inhibited the peptide transport of ^Tsn^TAP2/TAP1 (S3 Figure). Subsequently, we examined the effect of CPXV012 on 8-azido-ATP photo cross-linking of the TAP1/^Tsn^TAP2 complex. In the absence of CPXV012, TAP1 and TAP2 were specifically photo-labeled by 8-azido-ATP (Fig. 3B). In contrast, the TAP•^flag^CPXV012 complex showed a strongly reduced ATP photo labeling of TAP1 and TAP2. Taken together, these results suggest that CPXV012 inhibits ATP binding or the rate of nucleotide exchange of TAP1 and TAP2.

### US6 and BNLF2a prevent the formation of TAP•CPXV012 complexes

**Figure 4:**
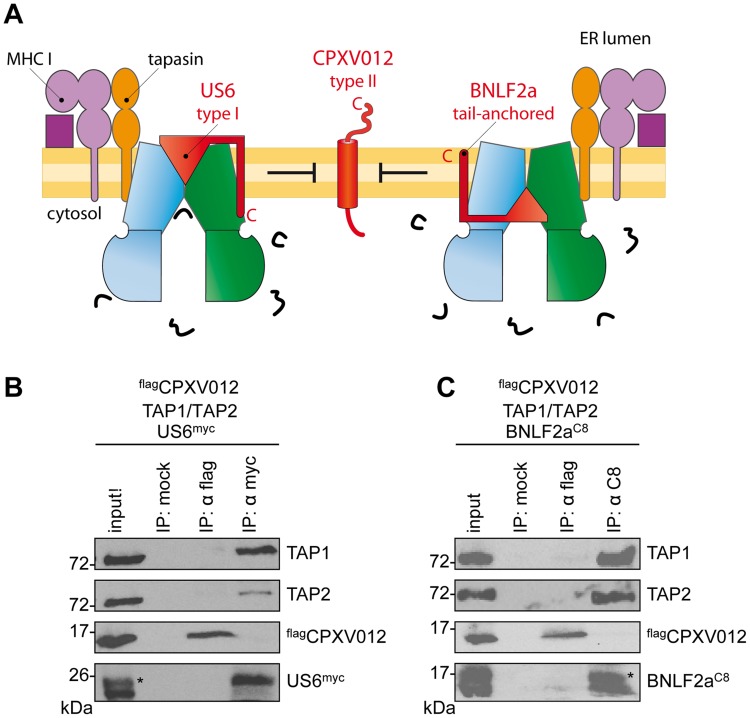
HCMV-US6 and EBV-BNLF2a prevent the formation of CPXV012•TAP complexes. (A) Schematic presentation of the MHC I peptide-loading complex and the viral immune evasin US6 (type I membrane protein), BNLF2a (tail-anchored), and CPXV012 (type II). (B, C) ^flag^CPXV012, TAP1, and TAP2 were coexpressed with either US6^myc^ (B) or BNLF2a^C8^ (C) in *Sf*9 cells. Proteins were affinity-purified with myc-, flag-, or C8-specific antibodies (IP). The HC10-antibody was used as negative control (mock). Samples were analyzed by immunoblotting with the corresponding antibodies. An aliquot (1/20) of the crude membrane input is shown. *, glycosylated protein.

We next investigated whether CPXV012 might recognize a similar TAP conformation as the immune evasin BNLF2a or US6, which target specific, yet different conformations of the antigen translocation complex [29]. After coexpression of TAP1, TAP2, ^flag^CPXV012, and either US6^myc^ (Fig. 4A and B) or BNLF2a^C8^ (Fig. 4A and C), coimmunoprecipitations were performed using antibodies against ^flag^CPXV012 (anti-flag), US6^myc^ (anti-myc), or BNLF2a^C8^ (anti-C8), respectively. Strikingly, expression of either US6 or BNLF2a prohibits binding of CPXV012 to TAP, while TAP1/2 was affinity purified with either US6^myc^ or BNLF2a^C8^. Moreover, if ^flag^CPXV012 was immunoprecipitated, almost no TAP was found to be associated with the cowpox viral factor, while TAP1/2•US6 or TAP1/2•BNLF2a complexes were detected. On the other hand, the formation of TAP1/2•CPXV012 complexes was not impaired in the absence of the viral TAP inhibitors (S4 Figure). Hence, US6 and BNLF2a prevent the formation of TAP1/2•CPXV012 complexes.

### A genomic frameshift causes TAP inhibition

**Figure 5:**
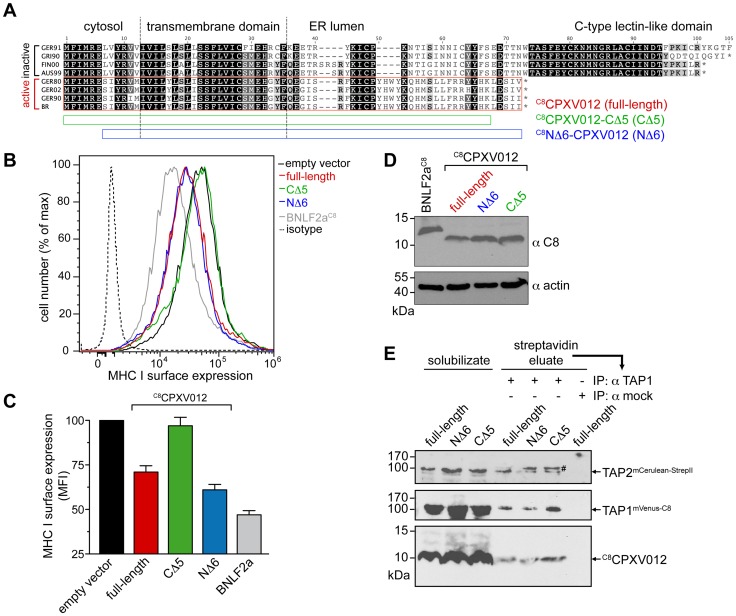
CPXV012 evolved a unique ER-lumenal sequence that is essential for TAP inhibition. (A) Sequence alignment of CPXV012 and its orthologs. Abbreviations and accession numbers are shown in Table S1. GER91: The first 97 amino acids of the protein are aligned. The CPXV012 sequence of Brighton Red strain was used in this study (red box). The N- and C-terminal deletion constructs ^C8^NΔ6-CPXV012 and ^C8^CPXV012-CΔ5 are indicated as blue and green bars. (B) The last C-terminal, ER-lumenal residues of CPXV012 are essential for TAP inhibition. HeLa cells were transiently transfected with empty vector, full-length ^C8^CPXV012, ^C8^CPXV012-CΔ5, ^C8^NΔ6-CPXV012, or BNLF2a^C8^ in pIRES2-EGFP, respectively. MHC I surface expression was analyzed by flow cytometry. Only GFP-positive cells were analyzed. The dotted histogram represents the isotype control. Histograms for mock, BNLF2a^C8^, ^C8^CPXV012 full-length, and isotype transfection are from the same data set in Fig. 1A. (C) Evaluation of the histograms from B. Mean fluorescence intensity (MFI) was calculated for cells transfected with the indicated constructs. (D) Similar expression levels of the ^C8^CPXV012 constructs and BNLF2a^C8^ in cells analyzed by flow cytometry were confirmed by anti-C8 and anti-actin immunoblotting. (E) Inactive ^C8^CPXV012-CΔ5 still binds to coreTAP1/2 heterodimers. Human coreTAP1^mVenus-C8^ and coreTAP2^mCerulean-StrepII^ were coexpressed in HEK293T cells together with the ^C8^CPXV012 variants as indicated. TAP1/2 heterodimeric complexes were tandem-affinity purified using streptavidin and anti-TAP1 (mAb 148.3) matrices. The HC10-antibody was used as negative control (mock). Input (solubilizate, 1/30 aliquot) and affinity purified complexes were analyzed by immunoblotting using C8- or TAP2-specific (mAb 435.3) antibodies, respectively. #, partially unfolded mCerulean.

A phylogenetic comparison revealed a substantial genotypic and phenotypic diversity among the twelve CPXV strains sequenced so far [34]. CPXV012 used in this study is derived from the reference strain “Brighton Red” (BR, Supplemental Table 1). Notably, all CPXV012 orthologs share 45 N-terminal residues, which include the cytosolic region of residue 1–12 and the transmembrane helix (aa 13–35) of the type II membrane protein (Fig. 5A). In contrast, the C-terminal regions are divergent and the CPXX012 orthologs cluster in two main groups. The first group, represented by strain GRI90 or GER91, contains proteins harboring an ER-lumenal domain of 50 to 100 residues, which encodes a C-type lectin-like domain (CTLD). These proteins do not suppress MHC I antigen processing [15]. In contrast, the ER-lumenal domains of CPXV012 belonging to the “Brighton Red” subgroup are significantly shorter and lack similarity to any known domain.

Why are the C termini of both subgroups distinct to each other? Surprisingly, if analyzed on the DNA level, all CPXV012 ORFs turned out to be very similar (S5 Figure). The ORFs of CPXV012 are localized in the terminal genomic region, where many non-essential genes are clustered that encode for proteins affecting virulence or interaction with the host immune system [35], [36]. Unlike the central region of the *Orthopoxvirus* genome, which is conserved and encodes proteins for virus replication, the terminal genomic regions are diverse and are more likely prone to DNA rearrangements or mutations. Indeed, the “Brighton Red” CPXV012 ORF differs by a deletion of five A-T pairs, thus causing a frameshift. The alternative reading frame starts right after the TMD and ends with a stop codon after 25 residues. Since these last C-terminal residues are unique, we hypothesize that this region may be functionally important. Hence, we generated CPXV012 variants lacking five or six amino acids at their C or N terminus (^C8^CPXV012-CΔ5 or ^C8^NΔ6-CPXV012, respectively). ^C8^NΔ6-CPXV012 suppressed the MHC I surface expression of HeLa cells to the same extent as full-length ^C8^CPXV012, whereas ^C8^CPXV012-CΔ5 was inactive (Fig. 5B and C), even though almost equivalent amounts of each CPXV012 variant were expressed (Fig. 5D). This lack of function was not due to an impaired interaction with TAP, because ^C8^CPXV012-CΔ5 can still interact with heterodimeric TAP1/2 complexes (Fig. 5E). Hence, the transmembrane region of CPXV012 is likely responsible for the interaction with TAP. Interestingly, the CPXV012 variant D10L, which contains a C-type lectin domain at its C-terminus and can therefore not inhibit TAP [15], interacts with the transporter (S6 Figure). This is due to the fact that both proteins share a high conserved N-terminal region including the transmembrane domain. In conclusion, the transmembrane domain mediates binding of the viral inhibitor to the TAP complex, whereas the last five C-terminal residues of CPXV012 are crucial for TAP inhibition.

### CPXV012 exploits a trans-inhibition mechanism

**Figure 6:**
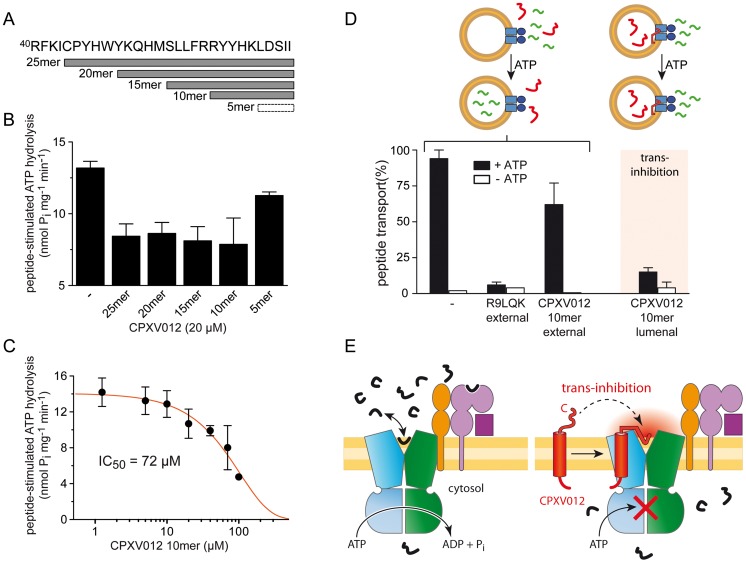
The ER-lumenal CPXV012 fragment is sufficient for trans-inhibition of antigen translocation. (A) CPXV012 sequence and synthetic peptides derived from the C-terminal ER-lumenal end of CPXV012. (B) The C-terminal 10 residues of CPXV012 are sufficient for inhibition of the peptide-stimulated ATP hydrolysis of TAP. Purified coreTAP (0.2 µM) was incubated with ATP (1 mM) and high-affinity substrate peptide R9LQK (1 µM) in the presence and absence of C-terminal CPXV012 peptides (20 µM) as indicated. Release of inorganic phosphate was quantified and normalized to the coreTAP-dependent hydrolysis in the presence of R9LQK. Each data point represents the mean of triplicate measurements. Error bars show S.D. (C) The ATP hydrolysis activity of purified TAP (0.2 µM) was measured in the presence of ATP (1 mM), substrate peptide R9LQK (0.5 µM), and increasing concentrations of CPXV012 10mer-peptide. By fitting of the data, a half-maximum inhibition value (IC_50_) of 72±20 µM was determined. (D) 10mer CPXV012 fragment blocks peptide translocation *in vitro*. Transport of RRYC^(F)^KSTEL peptide (1 µM; C^(F)^, fluorescein-labeled cysteine) by reconstituted coreTAP was analyzed in the presence and absence of ATP (3 mM) in combination with 100 µM unlabeled peptide R9LQK or CPXV012 10mer-peptide added to or entrapped in liposomes (external and lumenal, respectively). (E) Inhibition mechanism of CPXV012. TAP transports peptides until a critical concentration inside the ER lumen induces trans-inhibition of the transport complex.

Because this active C-terminal domain of CPXV012 is located within the ER lumen, these results further imply that CPXV012 inhibits ATP binding by TAP *via* an allosteric crosstalk across the ER membrane. Interestingly, the C terminus of CPXV012 exhibits all hallmarks of a high-affinity TAP substrate [37]: Two positive residues (Arg59 and Arg60), which correspond to positions one and two of a 11mer substrate peptide that ends with the hydrophobic Ile69 (S7 Figure). Strikingly, replacement of Arg59 and Arg60 by alanine (^C8^CPXV012^RR59AA^) diminished the ability of the viral inhibitor to suppress MHC I surface expression, because the peptide recognition motif of TAP within the C terminus of CPXV012 was abrogated (S7 Figure). Since ATP hydrolysis of TAP is tightly coupled to substrate binding and translocation [38], we assessed how the frameshifted C-terminal region of CPXV012 affects the peptide-stimulated ATPase activity of purified coreTAP complexes. Peptides representing the last 5, 10, 15, 20 or 25 residues of CPXV012 were synthesized with an acetylated N terminus (Fig. 6A). This modification is known to dramatically decrease the affinity for TAP avoiding that these CPXV012 fragments act as “classical” TAP substrates [37], [39]. The 25, 20, 15, and 10mer fragments significantly inhibited the peptide-stimulated ATP hydrolysis (Fig. 6B). The IC_50_ value for TAP inhibition by the 10mer CPXV012 fragment was 72±20 µM (Fig. 6C). This suggests that the C-terminal region of CPXV012 mimics a TAP substrate just before its release into the ER lumen, which induces the functional arrest of TAP by inhibiting ATP binding to the transport complex.

The direct proof of a novel trans-inhibition mechanism of CPXV012 was obtained by functional reconstitution of purified components into liposomes. After reconstitution, approximately half of the TAP complex face their NBD to the outside [40] and hence were exclusively energized by external ATP, thereby assuring uni-directional translocation of fluorescently labeled peptides into the lumen of the proteoliposomes [41]. Peptide transport was inhibited by competition with excess of unlabeled peptides as expected, but not by a 100-fold excess of the C-terminal CPXV012 fragment (Fig. 6D). This demonstrates that the active domain of CPXV012 is unable to block TAP from the cytosol. In contrast, encapsulation of the 10mer CPXV012 fragment into the proteoliposomes abolished peptide translocation by the TAP complex almost completely. Thus, the C-terminal fragment of CPXV012 blocks antigen translocation *in trans*. Since the tethered C-terminal peptide imitates a high-affinity peptide substrate, it is tempting to speculate that CPXV012 mechanistically exploits a novel trans-inhibition mechanism of the antigen processing machinery.

## Discussion

Virus spread is accomplished through subversion of antiviral host immunity by an arsenal of factors that target key molecular steps necessary to mount an immune response [42]. In particular, gene products encoded by *Herpesviridae* are known to attack multiple steps in MHC I antigen presentation, including the generation of peptides by the ubiquitin-proteasome pathway, translocation of peptides into the ER by TAP, and the mistargeting of MHC I [3], [43]. The poxvirus gene product CPXV012 was classified as an inhibitor of antigen delivery to MHC I [15], [16]. However, the direct target, the inhibition mechanism, and the evolutionary origin remained illusive.

By expressing CPXV012 in mammalian and insect cells, we establish that CPXV012 directly inhibits the coreTAP complex. Notably, CPXV012 interferes with ATP binding to TAP1 and TAP2 and hence peptide induced ATP hydrolysis *via* an allosteric crosstalk across the ER membrane. This is mediated by the C-terminal region of CPXV012 located in the ER lumen, at the opposite membrane side of the NBDs (Fig. 6E). Similar to US6 encoded by HCMV, CPXV012 does not influence peptide binding, but inhibits ATP binding to the TAP complex. However, besides using a related strategy, our results demonstrate that the inhibition mechanism of CPXV012 is unique and distinct from US6: US6 inhibits ATP binding to TAP1 [24], whereas CPXV012 blocks ATP binding or nucleotide exchange of both TAP subunits. In addition, CPXV012 acts as a small peptide, whereas the active region of US6 consists of a folded ER-lumenal domain of 125 residues. In conclusion, CPXV012 and US6 prevent ATP binding to TAP *via* two distinct mechanisms, which presents a striking example of convergent evolution. Since BNLF2a and US6, which target distinct conformation states of TAP [29], prevent CPXV012 binding to TAP, it is tempting to speculate that CPXV012 transiently intercepts the translocation cycle at a state, which is not accessible if TAP is arrested by either US6 or BNLF2a. However, we cannot exclude entirely that binding of either US6 or BNLF2a to TAP mask directly the CPXV012 interaction site on TAP due to steric competition.

In this study, we provide several lines of evidence that the active domain of the immune evasin is located at the C-terminal tip of CPXV012, which is tethered to the ER membrane and inhibits the coreTAP complex. First, a C-terminally deletion CPXV012-CΔ5 is inactive in blocking MHC I antigen processing, whereas an N-terminal deletion is active to the same extent as full-length CPXV012. Second, the ten C-terminal residues of CPXV012 are sufficient to inhibit the peptide-stimulated ATPase activity of coreTAP. Notably, inhibition by the 10mer fragment is compartment-specific, because it only arrests TAP from the lumen, not from the cytosol. This active C-terminal segment of CPXV012, which emerged from a frameshift in the CPXV012 gene, exhibits all hallmarks of a high-affinity TAP substrate: positively charged or aromatic residues at position 1–3 and a hydrophobic residue at its C terminus [37]. Disturbing this consensus sequence by replacing the N-terminal positively charged residues (Arg59 and Arg60) by an alanine (^C8^CPXV012^RR59AA^) rendered the viral inhibitor inactive. If combined, these results are in line with a recently discovered negative feedback mechanism of TAP (trans-inhibition) [41]: substrate peptides are transported against the gradient into the lumen of TAP-containing proteoliposomes until the transporter is inhibited in *trans* by accumulated lumenal peptides.

*In vivo*, a significant proportion of TAP-translocated peptides does not associate with MHC I molecules, since they are more selective in peptide binding, if compared to TAP [44]. Accumulation of non-optimal MHC I substrates may compete with newly transported peptides for MHC I loading. Moreover, high concentrations of ER-lumenal peptides might even induce the unfolded protein response (UPR) and ER stress as a direct consequence of peptide accumulation, or indirectly as a result of competition between peptides and proteins for folding, assembly and retrograde translocation [44]. There is growing evidence that the UPR intersects with the functioning of cells of the immune system [45]. For instance, ER stress induces a decrease of MHC I surface expression [46] and a reduction in TAP1 protein levels, which is caused by the expression of microRNA-346 that directly targets human TAP1 mRNA [47]. In summary, it seems reasonable to assume that a trans-inhibition mechanism of TAP, as identified recently [41], would prevent detrimental accumulation of peptides in the ER lumen and hence ER stress. In addition, trans-inhibition might synergistically cooperate with pathways that export TAP-imported peptides from the ER lumen [48], [49]. Since the C terminus of CPXV012 resembles a high-affinity TAP peptide substrate that is sufficient to inhibit the transporter as soluble lumenal peptide, we propose that CPXV012 exploits this trans-inhibition mechanism of the TAP machinery. The active C-terminal fragment of CPXV012 is membrane-anchored and bound to TAP *via* its TMD, thereby mimicking a high ER lumenal peptide concentration. This high local concentration may trigger the ER-lumenal low-affinity [41] peptide binding site of TAP, causing the inhibition of the transport complex *in trans* (Fig. 6E).

CPXV is the suggested ancestor of other orthopoxviruses and contains the most complete set of immune modulators [35]. They target host pathways that regulate immediate immune responses, particularly the IFNs, the chemokines, the pro-inflammatory cytokines, the complement system, and mediators that orchestrate adaptive immunity [50]. With regard to this repertoire of mechanisms, two immune evasins of CPXV, namely CPXV012 and CPXV203, are known to inhibit synergistically the MHC I antigen presentation pathway: As shown here, CPXV012 diminishes the peptide supply in the ER by exploiting a novel trans-inhibition mechanism, whereas CPXV203 interferes with trafficking of MHC I from the ER to the plasma membrane by reprogramming KDEL-receptor recycling [15], [16], [51]–[53].

Down-regulation of MHC I antigen presentation by CPXV012 and CPXV203 does not inhibit the priming of CPXV-specific CD8^+^ T lymphocytes (CTLs) during acute infection, but blocks these CTLs at the effector response level by preventing recognition of infected cells *in vivo* [54]. Since direct priming should be also inhibited by CPXV-induced MHC I down-regulation, these results supported a model in which cross-presentation is used for CD8^+^ T cell priming during CPXV infection [54]. On the other hand, MHC I down-regulation would also leave infected cells vulnerable to be killed by NK cells. Usually, mature NK cells are tolerated toward normal cells by integrating stimulatory and inhibitory signals transmitted *via* receptor-ligand interactions [55]. According to the “missing-self” hypothesis [56], NK cells recognize and eliminate cells that fail to express MHC I molecules. Hence, it is not surprising that orthopoxviruses encode immune evasins that inhibit NK-cell activation. For example, CPXV expresses the orthopoxvirus MHC class I-like protein OMCP [57], a high-affinity antagonist of the activating NK-receptor NKG2D [58]. An opposite strategy is the expression of decoy ligands for the NK cell inhibitory receptor NKR-P1B, which plays a critical role in the MHC I independent “missing self” recognition by detecting the C-type lectin-related ligand Clr-b [59], [60]. Indeed, rat cytomegalovirus expresses a C-type lectin-like (RCTL) gene product that resembles Clr-b, which inhibits NK killing of infected cells *via* direct interaction with NKR-P1B [7]. Interestingly, in a subgroup of CPXV strains, CPXV012 codes for D10L that contains the C-type lectin domain at the C terminus (Fig. 5A). D10L is highly homologous to Clr-b, but it is beyond the scope of this manuscript to determine whether D10L indeed functions as a decoy ligand for NKR-P1B. Nevertheless, D10L binds to the TAP complex, since the N-terminal cytosolic and transmembrane region of D10L and CPXV012 are highly conserved. The physiological relevance of this interaction remains to be determined. More importantly, D10L lacks the C-terminal alternative reading frame, which mimics a TAP substrate consensus sequence, and is therefore unable to inhibit MHC I surface presentation [15]. In combination with the fact that the transmembrane region of all CPXV012 variants mediate the interaction with TAP, their differences at the C terminus explains on a molecular level how a gene product adapts “incidentally” a new function, thereby crossing the frontier of sabotage and stealth.

While this manuscript was undergoing final revision, a report by Luteijn *et al.* was published that also describes data relevant to the viral immune evasions strategy of CPXV012 [61]. Consistent with our findings, it was shown that CPXV012 inhibits ATP binding, but not peptide binding to TAP. Substitutions of long polyalanine stretches within the ER-luminal region indicate that residues 41–65 of the viral protein are involved in TAP inhibition [61]. However, these experiments could not distinguish whether the loss-of-function is either caused because these long alanine stretches replace functionally important amino acids within the CPXV012 functional domain, or induce secondary effects, which then render the protein inactive. Here, we developed an opposite experimental strategy based on maintaining CPXV012 function. We show that the isolated C-terminal 10mer CPXV012 fragment is sufficient to inhibit ATPase activity of purified TAP. Moreover, by reconstitution of the inhibition pathway of CPXV012 by purified components, we provide direct evidence that this fragment is only active if provided *in trans*, on the ER-lumenal side of TAP. Based on these results, we conclude that the stretch of ten residues at the C terminus of CPXV012 is the active domain of the viral protein, which is necessary and sufficient to inhibit TAP. Interestingly, the ER-lumenal domain of CPXV012 displays a strong affinity for phospholipids and might reside at the lipid-water interface of the ER membrane in close proximity to the TMDs of TAP [61]. Since it is currently completely unknown where and how peptides are released form TAP inside the ER lumen, it is plausible that the low-affinity ER-lumenal peptide sensor [41], which initiates trans-inhibition, is located at the membrane-TMD interface of the translocation machinery.

Several key findings are disclosed by the present study. First, we decipher the molecular basis of how CPXV012 inhibits antigen presentation *via* MHC I by direct inhibition of ATP binding to coreTAP. Second, we show that the inhibition mechanism is distinct of all other yet identified viral TAP inhibitors. Thus, CPXV012 is an excellent tool to study the structure and function of TAP as well as new antigen processing pathways. Third, we propose that CPXV012 blocks TAP by hijacking the newly identified negative feedback mechanism of the antigen translocation machinery. Therefore, our findings provide an important tool to uncover the yet uncharacterized negative feedback regulation of TAP. Finally, by elucidating the functional evolution of the viral immune evasive protein CPXV012, this study provides the rare opportunity to decipher on a molecular level how nature plays hide and seek with a pathogen and its host.

## Materials and Methods

### Cloning, constructs and recombinant viruses

CPXV012 (Gene ID: 1485887) with an N-terminal His_10_-flag tag (^flag^CPXV012) was codon optimized for expression in mammalian and insect cells and synthesized *de novo* (GenScript, Piscataway, NJ, USA). ^flag^CPXV012 was cloned into pFastBac1 (Invitrogen, Carlsbad, CA, USA) vectors for the baculovirus production or was used as a template for PCR amplification. PCR reactions were performed under standard conditions using Phusion DNA polymerase (Finnzymes, Vantaa, Finland) and synthetic oligonucleotide primers (endonuclease cleavage sites are underlined). All constructs were verified by DNA sequencing. ^C8^CPXV012 was generated using C8-CPVX012-*Bam*HI-fo (GCGGATCC ATGCCGCGCGGCCCGGATCGCCCGGAAfGGCATTGAAGAATTCATCATGAGAGAGTCTATC), and CPXV012-*Eco*RI-rev (GCGAATTCTTAGATGATGCTATCCAGC) primers and was subcloned into pFASTBac1 (Invitrogen). Recombinant baculoviruses were generated using the Bac-to-Bac baculovirus expression system (Invitrogen).

For expression in human cells, PCR-generated products were cloned into pIRES2-EGFP (Clontech Laboratories Inc., Palo Alto, CA, USA) *via* the respective restriction sites upstream of the internal ribosome entry site (IRES) and enhanced GFP. ^C8^CPXV012, ^flag^CPXV012 (also containing an N-terminal His_10_-tag), and ^C8^CPXV012-CΔ5 were amplified with the following primers: C8-CPXV012-*Nhe*I-fo (AATGCTAGCACCATGGCA CCGCGCGGCCCGGATCGCC), Flag-CPXV012-*Nhe*I-fo (AATGCTAGCACCATGGCACACCAT CATCACCATCATCACCAC), CPXV012-full-length-*Bam*HI-rev (TTAGGATCCTCAGATGATG CTATCCAGCTTGTGGTAGTAC), and CPXV012-CΔ5-*Bam*HI-rev (TTAGGATCCTCACTTGTG GTAGTACCTGCGGAACAG), respectively. ^C8^NΔ6-CPXV012 was generated by three sequential PCR reactions using the overlapping forward primers CPXV012-NΔ6-PCR1-fo (CGGAAGGCATTGAAGAAAGCGGATCTATCTACCGTGTGATGATCG), CPXV012-PCR2-fo (CCGCGCGGCCCGGATCGCCCGGAAGGCATTGAAGAAAGCGGA), and C8-CPXV012-*Nhe*I-fo, and the reverse primer CPXV012-full-length-*Bam*HI-rev. Residues Arg59 and Arg60 of CPXV012 were simultaneously replaced by two alanines (^C8^CPXV012^RR59AA^) *via* site-directed mutagenesis of the template ^C8^CPXV012-pIRES2-EGFP using the primers CPXV012-R59AR60A-fo (GTCCCTGCTGTTCGCGGCATACTACCACAAGCTG) and CPXV012-R59A/R60A-rev (CAGCTTGTGGTAGTATGCCGCGAACAGCAGGGAC). D10L (Gene ID: 90660243) with an N-terminal C8 tag (^C8^D10L) was synthesized *de novo* and cloned into pIRES2-EGFP using *Nhe*I and *Bam*HI restriction sites. BNLF2a^C8-NST^-pIRES2-EGFP has been described previously [29]. Human coreTAP1^mVenus-His10^ (TAP1 residues 227-808; Q03518) and coreTAP2^mCerulean-StrepII^ (TAP2 residues 125-704; Q59H06) have been described previously [62].

### Cell lines, infection and transfection

*Spodoptera frugiperda (Sf9)* insect cells were grown in Sf900II medium (Invitrogen) following standard procedures. *Sf*9 cells were infected with recombinant baculovirus encoding for ^flag^CPXV012, ^C8^CPXV012, US6^myc^ [29], BNLF2a^C8-NST^ [29], codon-optimized human TAP1/2 [63], and human wild-type TAP1^His6^/TAP2 [64], coreTAP1/2 [11] respectively. 48 h after infection, cells were harvested, and membranes were prepared as described [64]. HeLa and HEK293T cells (ATCC CRL-11268) were cultured in DMEM (PAA Laboratories, Cölbe, Germany) supplemented with 10% fetal calf serum (FCS; Biochrom AG, Berlin, Germany) at 37°C in a 5% CO_2_-humidified atmosphere. For flow cytometry, HeLa cells (ATCC CCL-2) were seeded in 6-well plates with a density of 2×10^5^ cells/well the day before transfection. Transient transfection was conducted according to the Magnetofection protocol (Chemicell, Berlin, Germany) using 2 µg of plasmid DNA and 2 µl of PolyMAG (Chemicell) per well. For the tandem-affinity purification of TAP complexes, HEK293T cells were seeded in 150 mm dishes with a density of 2×10^6^ cells/dish. On the next day, cells were transfected using 90 µg of polyethyleneimine (Sigma-Aldrich, Taufkirchen, Germany) and 30 µg of plasmid DNA according to standard procedures.

### Antibodies and peptides

Monoclonal mouse anti-TAP1 (mAb 148.3) and anti-TAP2 (mAb 435.3) antibodies were used for immunoblotting and immunoprecipitation [64]. In certain experiments, polyclonal rabbit anti-TAP1 (1p2) and anti-TAP2 (2p4) antibodies were used for immunoprecipitation [65]. C8-tagged CPXV012 and BNLF2a were immunoprecipitated and detected by an anti-C8 antibody [66]. Flag-tagged CPXV012 was immunoprecipitated and detected by anti-flag antibodies purchased from Sigma-Aldrich (F1804). US6^myc^ was immunoprecipitated and detected by a monoclonal anti-myc antibody (4A6) from Millipore (Schwalbach, Germany). The HC10 antibody, which recognizes human MHC I heavy chain [67], was kindly provided by Hidde L. Ploegh (MIT, Cambridge, MA). Peptide-loaded MHC I molecules were detected by phycoerythrin (PE) coupled anti-human HLA-ABC (W6/32). Mouse IgG2a isotype control antibodies were purchased from BioLegend (San Diego, CA, USA). Actin was detected by a mouse anti-actin antibody purchased from Sigma-Aldrich (A2228). Peptides were prepared by solid-phase synthesis using the Fmoc/*t*Bu strategy. Peptides were purified by reverse-phase C_18_ HPLC, and their identity was confirmed by mass spectrometry. Peptides representing the last 25, 20, 15, 10, or 5 residues of CPXV012 were acetylated at their N termini.

### Flow cytometry

Transiently transfected HeLa cells were harvested 48 h after transfection and washed once with cold FACS buffer (PBS/2% FCS). After blocking with 5% BSA in FACS buffer on ice, cells were washed twice and incubated with W6/32-PE or the PE-coupled isotype control antibody for 15 min on ice in the dark. Cells were again washed twice and FACS analysis was performed using an Attune Acoustic Focusing Cytometer (Applied Biosystems, Life Technologies, Carlsbad, CA, USA). Data were analyzed with FlowJo software (TreeStar, Ashland, OR, USA). For evaluation, a FSC/SSC gate was drawn around the main cell population and only GFP-positive cells were analyzed with respect to MHC I surface expression. To confirm protein expression, parallel samples of transfected HeLa cells were solubilized in RIPA Lysis and Extraction Buffer (Thermo Fisher Scientific, Pierce, Rockford, IL, USA) and analyzed by SDS-PAGE (12%) and subsequent immunoblotting with anti-C8 antibodies.

### Immunoprecipitation and immunoblotting

Membranes prepared from insect cells (1 mg of total protein) were resuspended in 0.4 ml lysis buffer L1 (20 mM Tris/HCl, pH 7.5, 150 mM NaCl, 5 mM MgCl_2_, 2% (w/v) digitonin (Carl Roth, Karlsruhe), 1.5 mM phenylmethylsulfonylfluoride, 3 mM benzamidine). After solubilization for 60 min on ice, non-solubilized proteins were removed by centrifugation at 100,000× g for 30 min at 4°C. The supernatant was incubated with M-280 sheep anti-mouse IgG Dynabeads (Dynal Biotech, Hamburg), which had been pre-loaded with antibodies. Beads were washed three times with 1 ml of washing buffer (20 mM Tris/HCl, pH 7.5, 150 mM NaCl, 2 mM EDTA, 0.2% (w/v) digitonin). Proteins were eluted in 1× SDS sample buffer (2% SDS, 50 mM Tris/HCl, pH 8.0, 200 mM DTT, 10% glycerol, 0.05% bromophenol blue) for 3 min at 65°C. Samples were denatured for 20 min at 65°C and separated by SDS-PAGE (12% or 6%). After electro transfer onto nitrocellulose membranes, proteins were detected with specific antibodies as indicated. Horseradish peroxidase (HP) conjugated secondary antibodies were detected with Lumi-Imager F1 (Roche). One representative blot out of three independent experiments is shown.

### Peptide transport

Membranes prepared from insect cells (0.1 mg of total protein) were resuspended in 50 µl of AP buffer (5 mM MgCl_2_ in PBS, pH 7.4) in the presence of 3 mM ATP. The transport reaction was started by adding 1 µM of the peptide RRYQNSTC^(F)^L (C^(F)^, fluorescein-labeled cysteine) for 3 min at 32°C and terminated with 1 ml ice-cold stop buffer (10 mM EDTA in PBS, pH 7.0). After centrifugation at 20,000× g for 8 min, the pellet was solubilized in 0.5 ml of lysis buffer L2 (50 mM Tris/HCl pH 7.5, 150 mM NaCl, 5 mM KCl, 1 mM CaCl_2_, 1 mM MnCl_2_, 1% Nonidet P-40) for 30 min at 4°C. Non-solubilized proteins were removed by centrifugation and the supernatant was incubated with 60 µl ConA Sepharose (50% w/v, Sigma-Aldrich) for 1 h at 4°C. After three washing steps with 0.5 ml lysis buffer each, ConA-bound peptides were specifically eluted with methyl-α-D-mannopyranoside (200 mM) and quantified by a fluorescence plate reader (λ_ex/em_ 485/520 nm; Polarstar Galaxy, BMG Labtech, Offenburg, Germany). Background transport was determined in the presence of apyrase (1 U/sample). All measurements were performed in triplicate.

### Peptide binding

Membranes prepared from insect cells (0.1 mg of total protein) were incubated with 0.5 µM of the peptide RRYC^(F)^KSTEL (C^(F)^, fluorescein-labeled cysteine) in 50 µl of AP buffer for 15 min at 4°C. Free peptides were removed by washing the membranes twice with 1 ml of ice-cold AP buffer and subsequent centrifugation at 20,000× g for 8 min. Membranes were lysed with 0.3 ml AP buffer containing 1% SDS, and peptides were quantified by a fluorescence plate reader. All measurements were performed in triplicate. Background binding was determined in 100-fold excess of unlabeled high-affinity substrate peptide R9LQK (RRYQKSTEL).

### Chemical cross-linking

Membranes prepared from insect cells (0.5 mg of total protein) were resuspended in 100 µl of ice-cold PBS buffer and incubated with the homobifunctional cross-linker ethylene glycol bis(succinimidyl succinate) (EGS, Thermo Scientific, Rockford, IL) at a final concentration of 0.5 mM. After incubation for 30 min at 4°C, the reaction was stopped by adding Tris/HCl buffer, pH 7.5 (50 mM final concentration). Membranes were collected by centrifugation (20,000× g for 8 min at 4°C) and analyzed by SDS-PAGE (6%) and immunoblotting using TAP1 or TAP2 specific antibodies. As specifically indicated, membranes were pre-incubated with 10 µM peptide R9LQK at 4°C for 1 h prior to cross-linking. One representative blot out of three independent experiments is shown.

### 8-Azido-ATP photo cross-linking

TAP1/2 complexes were purified from *Sf*9 membranes (600 µg of total protein) by immunoprecipitation using anti-TAP1 (mAb 148.3) or anti-C8 antibodies as described above. Dynabead immobilized complexes were pre-incubated in ATP binding buffer (20 mM HEPES, pH 7.4, 137 mM NaCl, 3 mM MgCl_2_) containing 15 µM of 8-azido-ATP[γ]biotin (Biolog Life Science Institute, Bremen, Germany) for 5 min on ice. Controls contained 15 µM 8-azido-ATP[γ]biotin and 5 mM unlabeled ATP. Photo cross-linking was initiated by UV irradiation (254 nm hand-held UV lamp) for 5 min on ice. After three washing steps in ATP-binding buffer, samples were denatured for 20 min at 65°C and separated by SDS-PAGE (12%). After electro transfer onto nitrocellulose membranes, proteins were detected with specific antibodies as indicated. Biotinylated proteins were visualized using an extravidin-HRP conjugate. Alternatively, membranes prepared from insect cells (600 µg of total protein) were resuspended in 150 µl ATP binding buffer and pre-incubated with 15 µM of 8-azido-ATP[γ]biotin in the presence and absence of an excess of ATP (5 mM) for 5 min on ice. After subsequent photo cross-linking for 5 min on ice, membranes were collected by sedimentation (20,000× g for 8 min), washed three times in ATP binding buffer, and then solubilized in 300 µl lysis buffer L1. Immunoprecipitations were performed using antibodies against either TAP1 (mAb 148.3) or the C8 tag as described above. Samples were analyzed by SDS-PAGE (12%) and subsequent immunoblotting with extravidin-HRP or the corresponding antibodies. One representative blot out of three independent experiments is shown.

### Isolation of TAP complexes by tandem-affinity purification

Since overexpressed TAP1 or TAP2 subunits might form homodimers, a orthogonal purification strategy was applied that ensures the isolation of heterodimeric TAP1/2 complexes [62]. TAP1/2-expressing HEK293T cells were resuspended in ice-cold buffer A (50 mM Tris/HCl, pH 8.0, 250 mM NaCl, 10% glycerol, 0.05% (w/v) digitonin) supplemented with 1% (w/v) digitonin and incubated for 1 h in an overhead shaker. All steps were carried out at 4°C. Non-solubilized proteins were removed by centrifugation at 150,000× g for 1 h at 4°C. Proteins were bound to 100 µl streptavidin agarose beads (Pierce, Rockford, IL, USA) for 1 h. The beads were washed two times with 1 ml buffer A. Proteins were eluted with 1 ml buffer A supplemented with 2.5 mM biotin. The streptavidin agarose eluate was incubated for 1 h with IgG Dynabeads, which had been pre-loaded with anti-TAP1 antibodies (mAb 148.3). After three washing steps, the affinity-purified protein was eluted with 2× SDS buffer for 30 min at 37°C and analyzed by SDS-PAGE and immunoblotting.

### Cell-free translation in rabbit reticulocyte lysate

To prepare coreTAP-containing insect ER microsomes, *Sf*9 cells were infected with recombinant baculovirus encoding for coreTAP1/2 [11]. 48 h after infection, cells were harvested, and membranes were prepared as described [68]. For the generation of mRNAs, ^C8^CPXV012 or ^C8^D10L was amplified directly from the corresponding pIRES2-EGFP plasmid using SP6-pIRES-fo (GATTTAGGTGACACTATAGAATACCACCGTCTATA TAAGCAGAGCTGGTTTAGTGAACC) and pIRES-GFP-rev (GTTTACGTCGCCGTCCAGC) primers. Purified PCR product was transcribed *in vitro* using SP6 RNA polymerase as before [29]. *In vitro* translation of purified mRNA (typically 50 µl reactions, 30°C, 40 min) was performed in the presence of rabbit reticulocyte lysate (Promega, Madison, WI, USA), *Sf*9 microsomes, and [^35^S]Met (0.4 µCi/µl). After translation, samples were analyzed by immunoprecipitation and phosphoimaging.

### ATP hydrolysis activity of purified TAP

Peptide stimulated ATPase activity was determined by a colorimetric assay based on the complex formation of free inorganic phosphate and ammonium molybdate with malachite green [69]. CoreTAP1^mVenus-His10^ and coreTAP2^mCerulean-StrepII^ were expressed in *Pichia pastoris* and solubilized in 2% (w/v) digitonin as described previously [40], [62]. CoreTAP1/2 heterodimers were purified by an orthogonal strategy using metal-affinity chromatography and subsequent streptactin-affinity chromatography as described [62]. For ATP hydrolysis measurements, 0.2 µM of purified coreTAP1/2 was incubated with 1 mM MgATP and the high-affinity substrate peptide R9LQK (1.0 µM) for 30 min at 37°C as described previously [63].

### Peptide translocation into proteoliposomes

For reconstitution of purified coreTAP1/2 into liposomes, large unilamellar vesicles consisting of *Escherichia coli* polar lipid extract (Avanti Polar Lipids Inc., Alabaster, AL, USA) and 1,2-dioleoyl-*sn*-glycero-3-phosphocholine (Avanti Polar Lipids, Inc.) with a molar ratio of 7∶3 were prepared as previously [63]. Detergent-destabilized vesicles and purified coreTAP1/2 were mixed with a lipid to protein ratio of 20∶1 (w/w) and incubated for 30 min at 4°C. Detergent was removed by incubation with polystyrene beads as described previously [40]. Proteoliposomes were pelleted for 30 min at 80,000 rpm at 4°C. Protein aggregates, empty vesicles and proteoliposomes were separated by centrifugation on a continuous Ficoll density gradient [40]. After washing, proteoliposomes were resuspended to a final concentration of 5 mg/ml in reaction buffer (20 mM HEPES, 200 mM NaCl, 50 mM KCl, 5% glycerol, pH 7.3). For peptide transport, coreTAP1/2 (0.5 µg) containing proteoliposomes were pre-incubated on ice in 45 µl reaction buffer containing 1 µM fluorescence labeled peptide RRYC^(F)^KSTEL. Transport was started by adding 3 mM MgATP and performed for 10 min at 37°C. The reaction was stopped as described previously [63]. For encapsulation of peptides, TAP proteoliposomes in reaction buffer containing the active CPXV012 fragment were snap frozen in liquid nitrogen and thawed on ice. After three freeze and thaw cycles, proteoliposomes were washed twice to remove the remaining, not encapsulated peptide. Peptide transport assay was performed as described above.

## Supporting Information

S1 Figure **Membranes express similar amounts of TAP1 and TAP2.** TAP1, TAP2, ^flag^CPXV012, and BNLF2a^C8^ were coexpressed in *Sf*9 insect cells as indicated. Equal amounts of crude membranes used for the peptide transport (Figure 2A) and peptide binding (Figure 2B) assays were analyzed by SDS-PAGE (12%) and immunoblotting with either anti-flag, anti-C8, monoclonal anti-TAP1 (mAb 148.3), or anti-TAP2 (mAb 435.3) antibodies. The double band represents glycosylated and non-glycosylated BNLF2a^C8^.
(TIF)

S2 Figure **CPXV012 inhibits peptide transport but not peptide binding to coreTAP.** ^flag^CPXV012 was coexpressed with coreTAP1/2 in *Sf*9 cells. (A) CPXV012 inhibits peptide transport of coreTAP. Crude membranes were incubated with RRYQNSTC^(F)^L peptide (C^(F)^, fluorescein-labeled cysteine) in the presence and absence of ATP. Transported and N-core glycosylated peptides were bound to ConA-beads and quantified by fluorescence. Peptide transport by TAP was set to 100%. The means of at least three independent experiments are shown. Error bars indicate the S.D. (B) CPXV012 does not inhibit peptide binding to coreTAP. Crude membranes were incubated with RRYC^(F)^KSTEL peptide (filled bars). A 100-fold excess of R9LQK was used to probe for unspecific binding (open bars). Membrane-associated peptide was quantified by fluorescence. (C) Membranes used for the peptide transport/binding assays express similar amounts of coreTAP1/2. Equal amounts of crude membranes were analyzed by SDS-PAGE (12%) and immunoblotting with the corresponding antibodies.
(TIF)

S3 Figure **CPXV012 inhibits peptide translocation of TAP1/^Tsn^TAP2 complexes.** ^flag^CPXV012 was coexpressed with TAP1 and ^Tsn^TAP2 in *Sf*9 cells. Crude membranes were incubated with RRYQNSTC^(F)^L peptide (C^(F)^, fluorescein-labeled cysteine) in the absence or presence of MgATP. N-core glycosylated and thus translocated peptides were bound to ConA-beads and quantified by fluorescence. Peptide transport by TAP was set to 100%. The means of at least three independent experiments are shown. Error bars indicate the S.D. (B) Membranes used for the peptide transport assay express similar amounts TAP1/^Tsn^TAP2. Equal amounts of crude membranes were analyzed by SDS-PAGE (12%) and immunoblotting with the corresponding antibodies.
(TIF)

S4 Figure **HCMV-US6 prevents the formation of CPXV012/TAP complexes.** ^flag^CPXV012, TAP1, and TAP2 were coexpressed with or without US6^myc^ in *Sf*9 cells. Proteins were affinity-purified with TAP1-, flag-, or myc-specific antibodies (IP). The HC10-antibody was used as negative control (mock). Samples were analyzed by immunoblotting with the corresponding antibodies. An aliquot (1/20) of the crude membrane input (input) is shown.
(TIF)

S5 Figure **Amino acid (A) and nucleotide (B) alignment of CPXV012 orthologs.** Abbreviations and accession numbers are shown in Supplemental Table 1. (A) GER91: The N-terminal 97 residues of the protein are aligned. The CPXV012 sequence of CPXV strain Brighton Red (box) was used in this study.
(TIF)

S6 Figure **Interaction of CPXV012 and its C-type lectin containing homolog D10L with TAP.** CPXV012 and D10L were *in vitro* translated in presence of coreTAP containing *Sf*9 derived ER microsomal membranes. Proteins were immunoprecipitated with a combination of TAP1 (mAb 148.3) and TAP2- (mAb 435.3) specific antibodies (IP TAP). The HC10-antibody was used as negative control (IP mock). Samples were analyzed by SDS-PAGE (10%) and subsequent phosphoimaging. An aliquot (1/20) of the *in vitro* translation reaction as input is shown. #, unspecific translation products.
(TIF)

S7 Figure **The C terminus of CPXV012 resembles a TAP substrate peptide.** (A) Comparison of the TAP binding motif and the C terminus (residues 55–69) of CPXV012. Favored residues of TAP are given at the individual positions as extracted using combinatorial peptide libraries. (B) Residues Arg59 and Arg60 of CPXV012 are essential for TAP inhibition. HeLa cells were transiently transfected with empty vector, full-length ^C8^CPXV012, ^C8^CPXV012^RR59AA^, or ^C8^CPXV012-CΔ5 in pIRES2-EGFP, respectively. MHC I surface expression of GFP-positive cells was analyzed by flow cytometry. (C) Mean fluorescence intensities (MFI) were calculated for cells transfected with the indicated constructs. (D) Expression levels of the ^C8^CPXV012 constructs in cells analyzed by flow cytometry were confirmed by anti-C8 and anti-actin immunoblotting.
(TIF)

S1 Table **CPXV012 orthologs.**
(DOCX)
