# Supplementary figures and images for: A Negative Feedback Modulator of Antigen Processing Evolved from a Frameshift in the Cowpox Virus Genome
Source: PLoS Pathog. 2014 Dec 11;10(12):e1004554. doi: 10.1371/journal.ppat.1004554 (PMC4263761; doi:10.1371/journal.ppat.1004554)

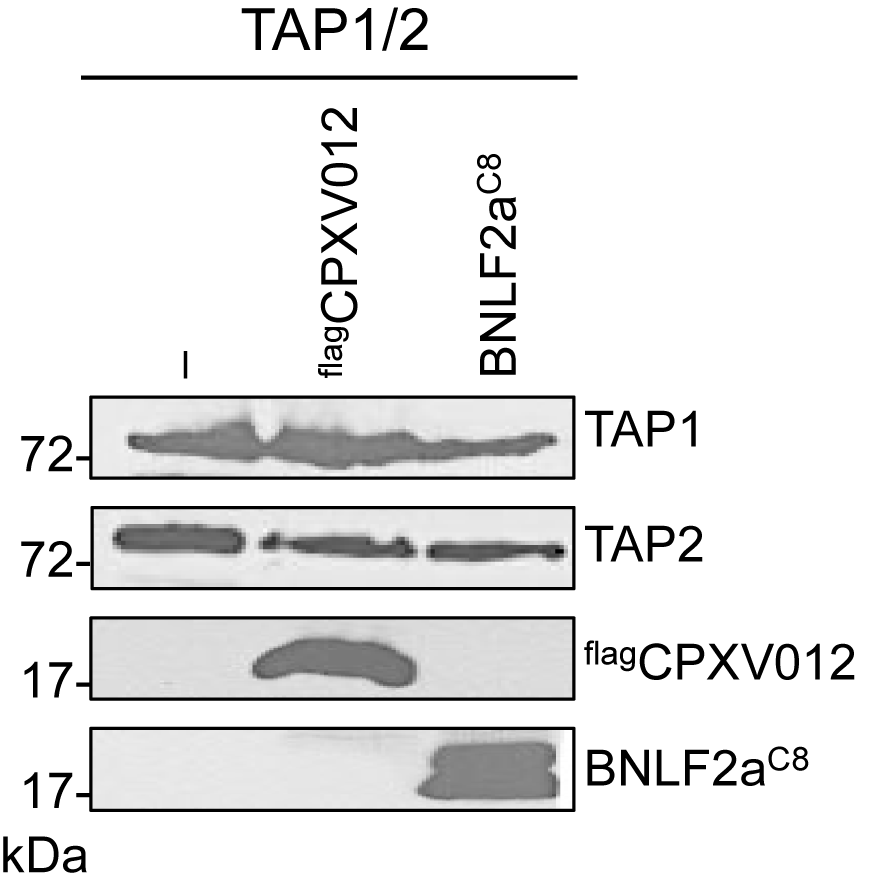

Supplement: S1 Figure — Membranes express similar amounts of TAP1 and TAP2. TAP1, TAP2, flagCPXV012, and BNLF2aC8 were coexpressed in Sf9 insect cells as indicated. Equal amounts of crude membranes used for the peptide transport (Figure 2A) and peptide binding (Figure 2B) assays were analyzed by SDS-PAGE (12%) and immunoblotting with either anti-flag, anti-C8, monoclonal anti-TAP1 (mAb 148.3), or anti-TAP2 (mAb 435.3) antibodies. The double band represents glycosylated and non-glycosylated BNLF2aC8. (TIF) [file ppat.1004554.s001.tif]

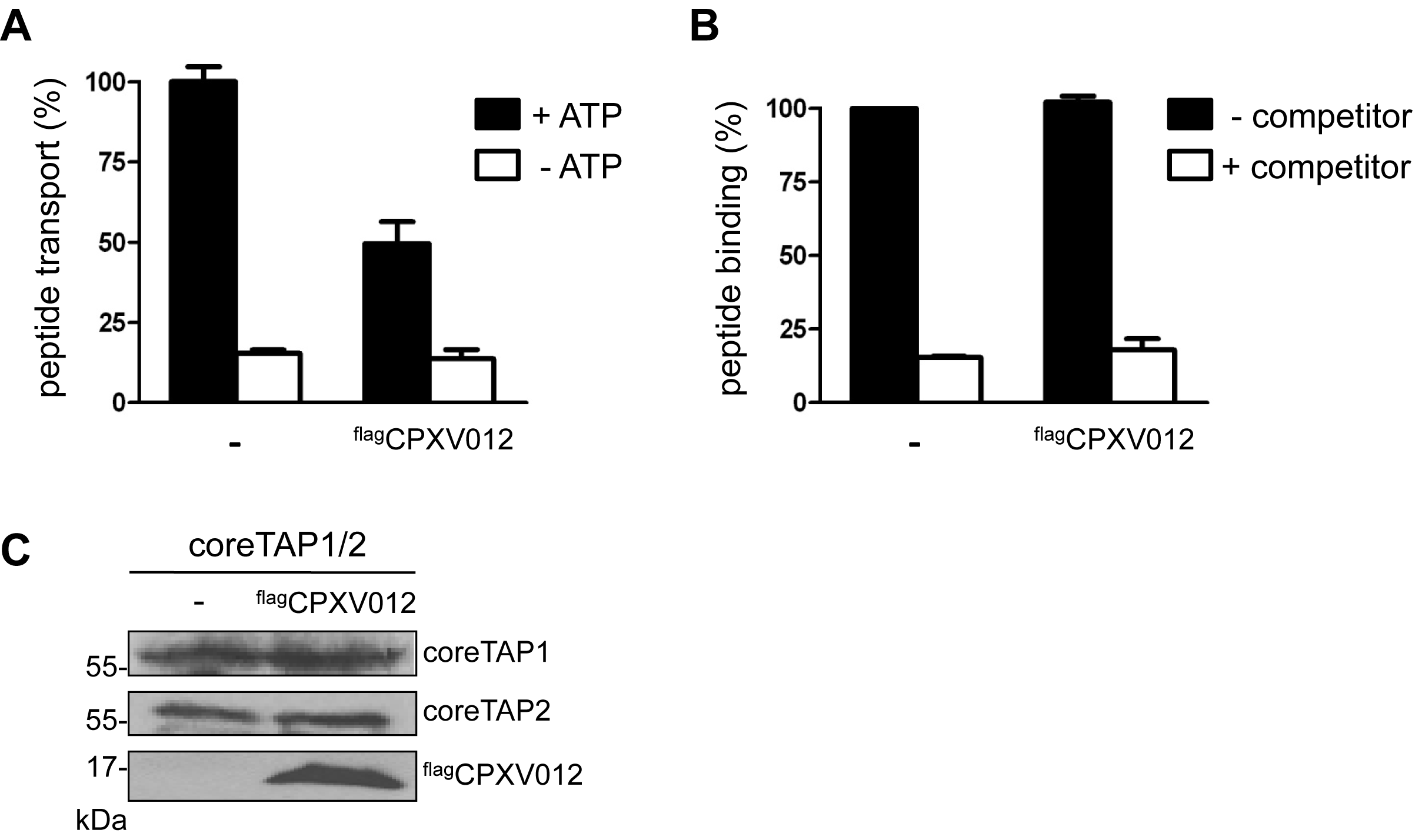

Supplement: S2 Figure — CPXV012 inhibits peptide transport but not peptide binding to coreTAP. flagCPXV012 was coexpressed with coreTAP1/2 in Sf9 cells. (A) CPXV012 inhibits peptide transport of coreTAP. Crude membranes were incubated with RRYQNSTC(F)L peptide (C(F), fluorescein-labeled cysteine) in the presence and absence of ATP. Transported and N-core glycosylated peptides were bound to ConA-beads and quantified by fluorescence. Peptide transport by TAP was set to 100%. The means of at least three independent experiments are shown. Error bars indicate the S.D. (B) CPXV012 does not inhibit peptide binding to coreTAP. Crude membranes were incubated with RRYC(F)KSTEL peptide (filled bars). A 100-fold excess of R9LQK was used to probe for unspecific binding (open bars). Membrane-associated peptide was quantified by fluorescence. (C) Membranes used for the peptide transport/binding assays express similar amounts of coreTAP1/2. Equal amounts of crude membranes were analyzed by SDS-PAGE (12%) and immunoblotting with the corresponding antibodies. (TIF) [file ppat.1004554.s002.tif]

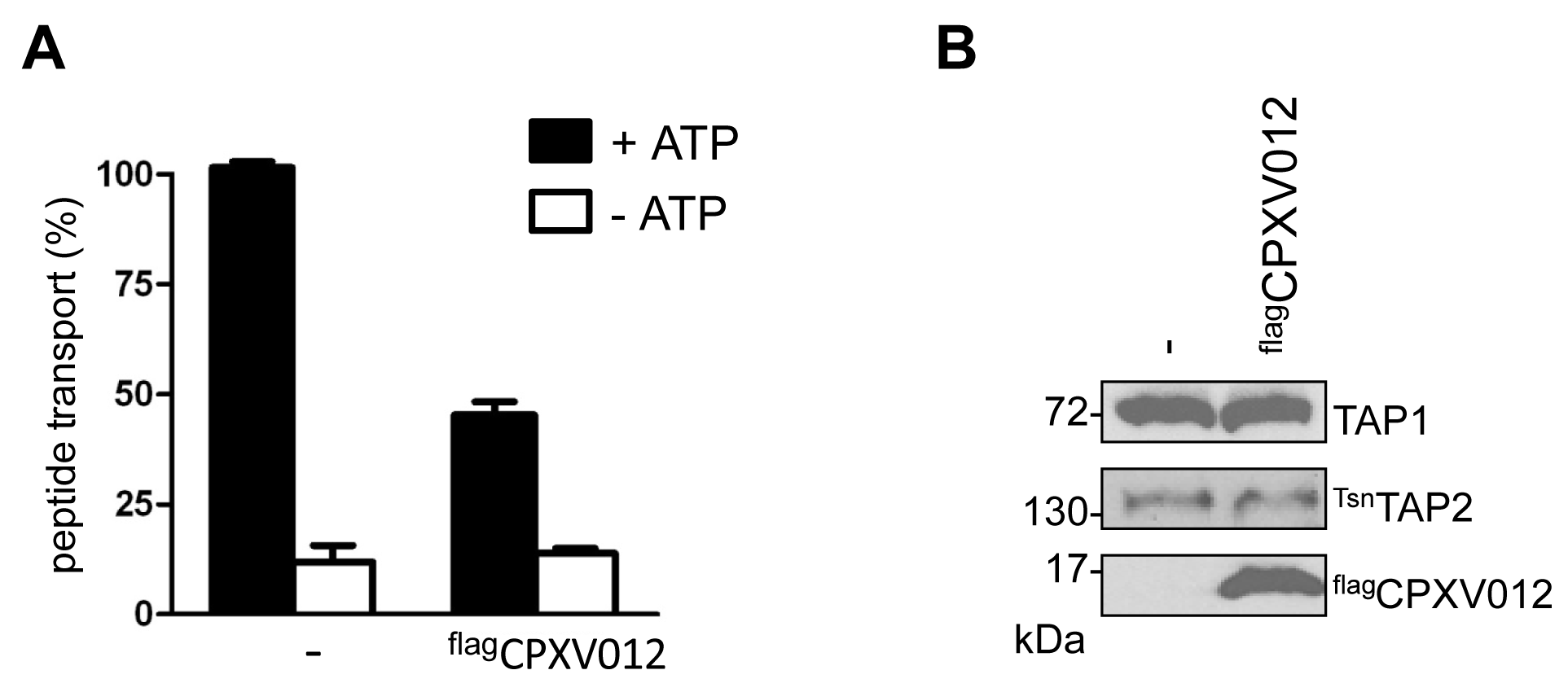

Supplement: S3 Figure — CPXV012 inhibits peptide translocation of TAP1/TsnTAP2 complexes. flagCPXV012 was coexpressed with TAP1 and TsnTAP2 in Sf9 cells. Crude membranes were incubated with RRYQNSTC(F)L peptide (C(F), fluorescein-labeled cysteine) in the absence or presence of MgATP. N-core glycosylated and thus translocated peptides were bound to ConA-beads and quantified by fluorescence. Peptide transport by TAP was set to 100%. The means of at least three independent experiments are shown. Error bars indicate the S.D. (B) Membranes used for the peptide transport assay express similar amounts TAP1/TsnTAP2. Equal amounts of crude membranes were analyzed by SDS-PAGE (12%) and immunoblotting with the corresponding antibodies. (TIF) [file ppat.1004554.s003.tif]

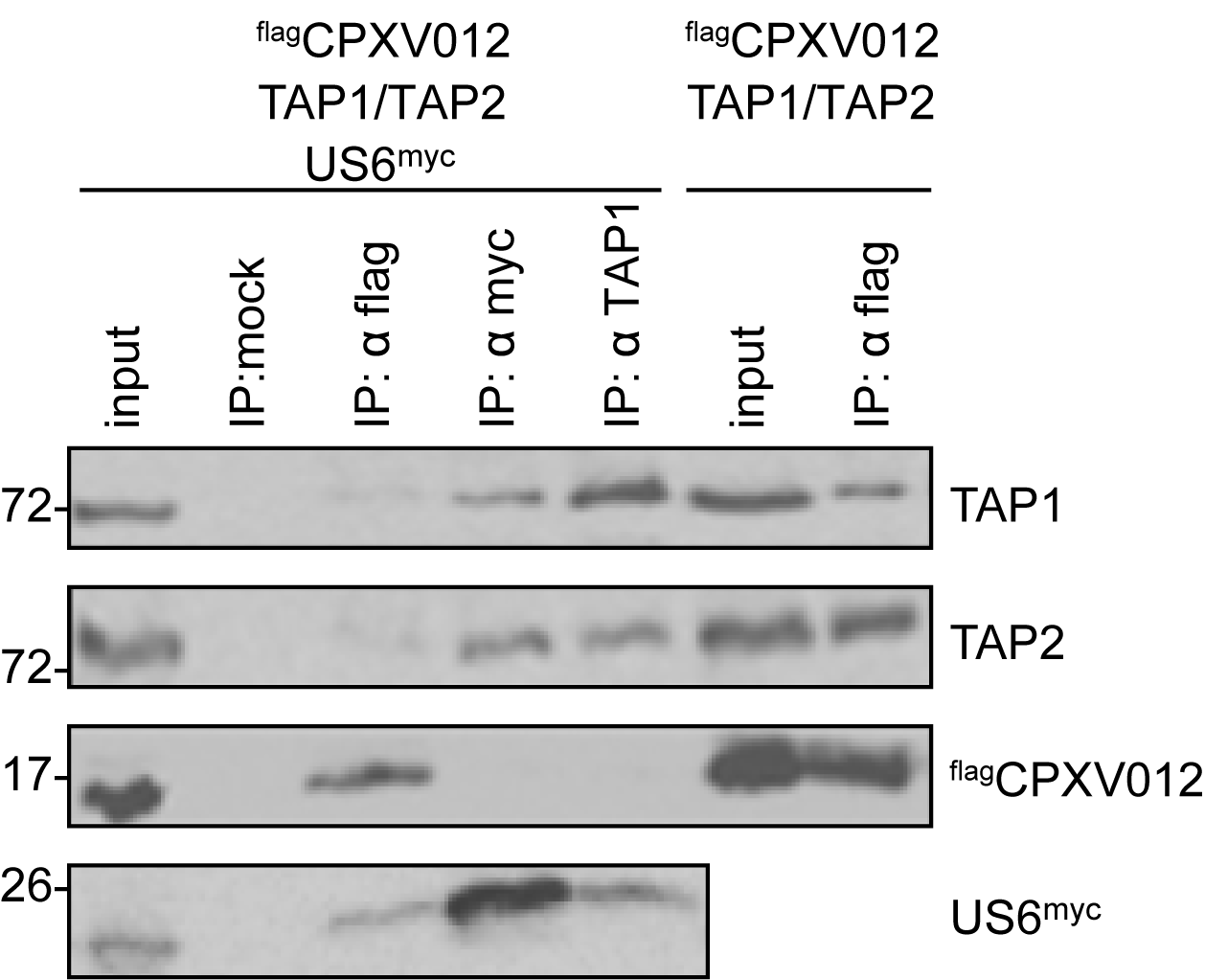

Supplement: S4 Figure — HCMV-US6 prevents the formation of CPXV012/TAP complexes. flagCPXV012, TAP1, and TAP2 were coexpressed with or without US6myc in Sf9 cells. Proteins were affinity-purified with TAP1-, flag-, or myc-specific antibodies (IP). The HC10-antibody was used as negative control (mock). Samples were analyzed by immunoblotting with the corresponding antibodies. An aliquot (1/20) of the crude membrane input (input) is shown. (TIF) [file ppat.1004554.s004.tif]

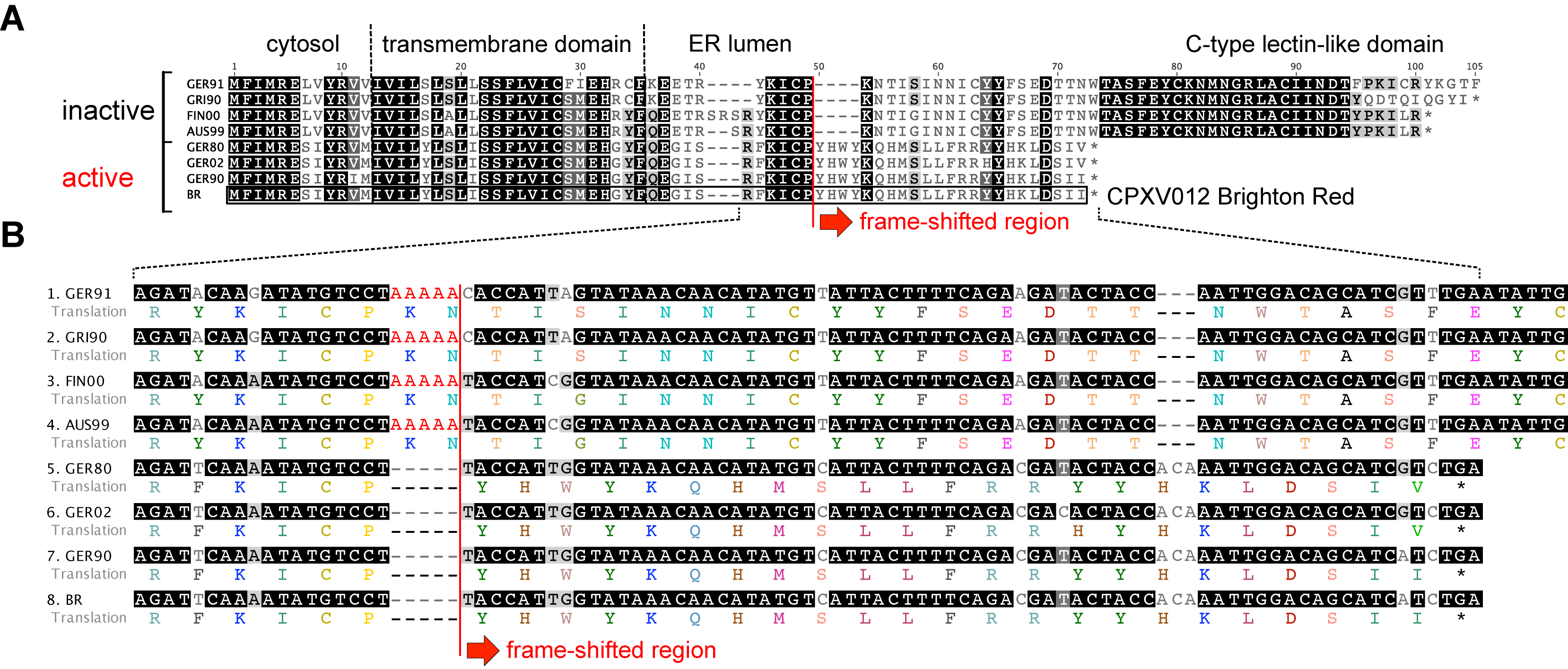

Supplement: S5 Figure — Amino acid (A) and nucleotide (B) alignment of CPXV012 orthologs. Abbreviations and accession numbers are shown in Supplemental Table 1. (A) GER91: The N-terminal 97 residues of the protein are aligned. The CPXV012 sequence of CPXV strain Brighton Red (box) was used in this study. (TIF) [file ppat.1004554.s005.tif]

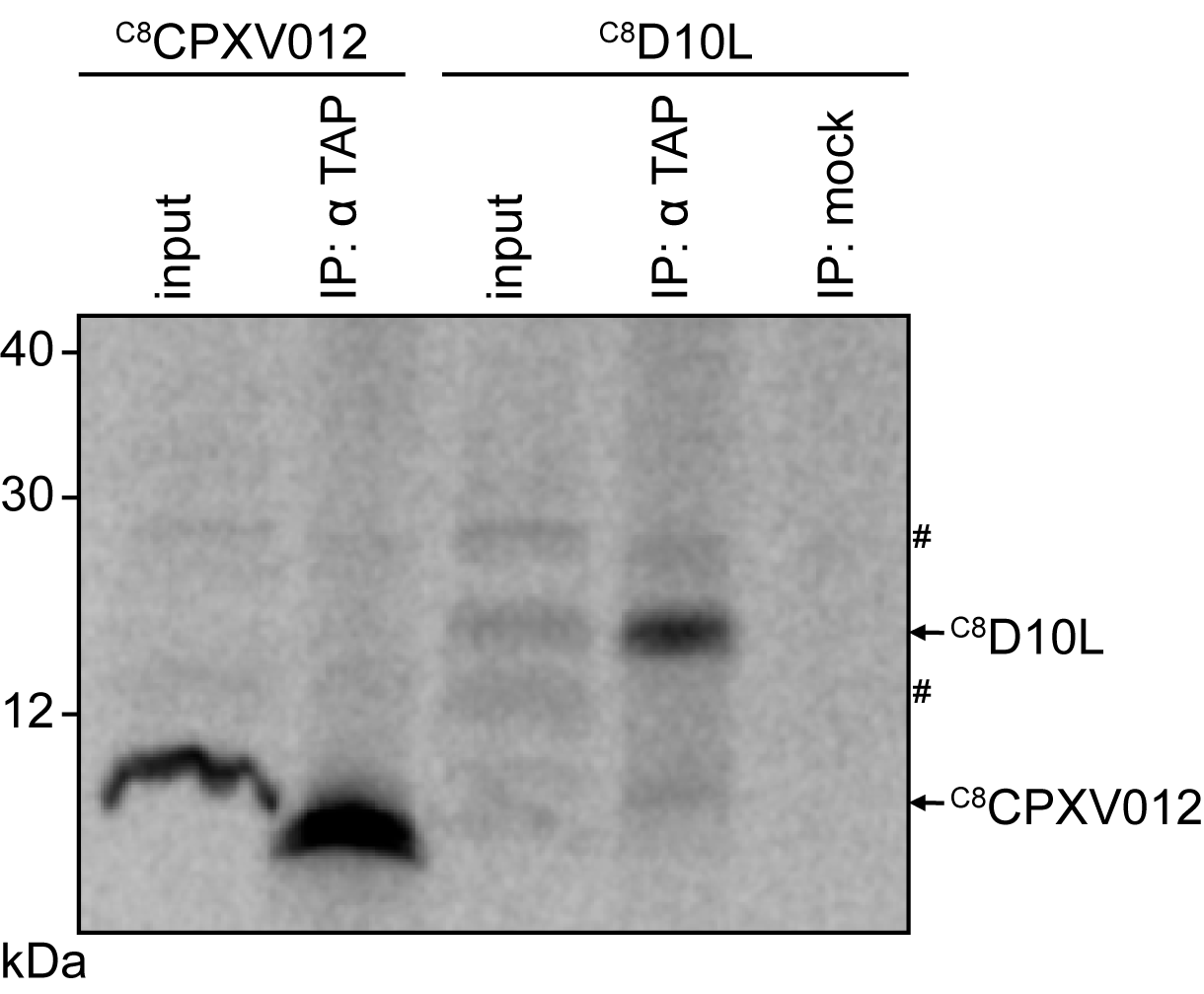

Supplement: S6 Figure — Interaction of CPXV012 and its C-type lectin containing homolog D10L with TAP. CPXV012 and D10L were in vitro translated in presence of coreTAP containing Sf9 derived ER microsomal membranes. Proteins were immunoprecipitated with a combination of TAP1 (mAb 148.3) and TAP2- (mAb 435.3) specific antibodies (IP TAP). The HC10-antibody was used as negative control (IP mock). Samples were analyzed by SDS-PAGE (10%) and subsequent phosphoimaging. An aliquot (1/20) of the in vitro translation reaction as input is shown. #, unspecific translation products. (TIF) [file ppat.1004554.s006.tif]

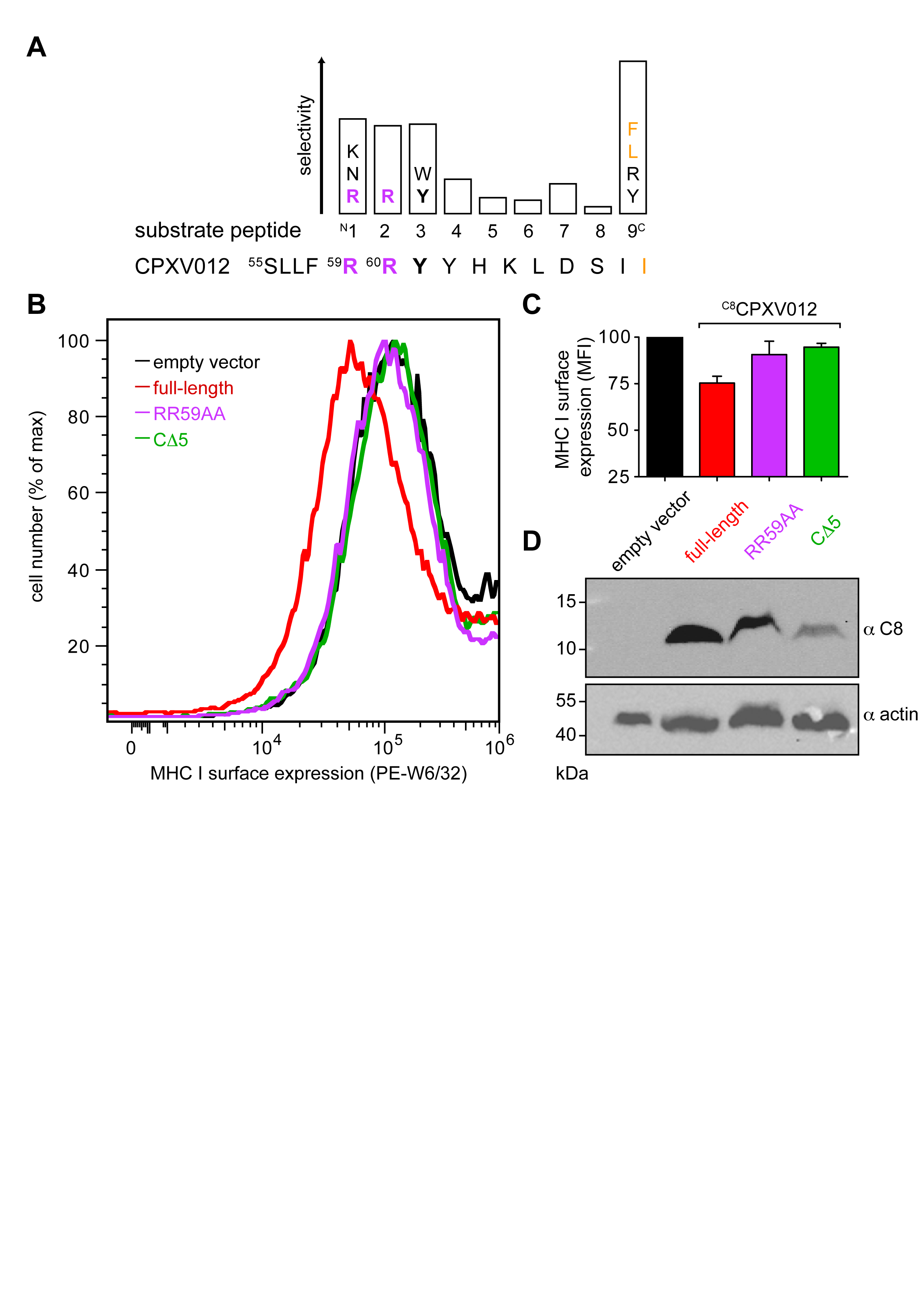

Supplement: S7 Figure — The C terminus of CPXV012 resembles a TAP substrate peptide. (A) Comparison of the TAP binding motif and the C terminus (residues 55–69) of CPXV012. Favored residues of TAP are given at the individual positions as extracted using combinatorial peptide libraries. (B) Residues Arg59 and Arg60 of CPXV012 are essential for TAP inhibition. HeLa cells were transiently transfected with empty vector, full-length C8CPXV012, C8CPXV012RR59AA, or C8CPXV012-CΔ5 in pIRES2-EGFP, respectively. MHC I surface expression of GFP-positive cells was analyzed by flow cytometry. (C) Mean fluorescence intensities (MFI) were calculated for cells transfected with the indicated constructs. (D) Expression levels of the C8CPXV012 constructs in cells analyzed by flow cytometry were confirmed by anti-C8 and anti-actin immunoblotting. (TIF) [file ppat.1004554.s007.tif]
