# Supplementary material for: A Negative Feedback Modulator of Antigen Processing Evolved from a Frameshift in the Cowpox Virus Genome
Source: PLoS Pathog. 2014 Dec 11;10(12):e1004554. doi: 10.1371/journal.ppat.1004554 (PMC4263761; doi:10.1371/journal.ppat.1004554)
Supplement: S1 Table — CPXV012 orthologs. (DOCX) [file ppat.1004554.s008.docx]

**Table S1: CPXV012 orthologs**

| Abbreviation | CPXV Strain | Length (aa) | Definition | Accession |
| --- | --- | --- | --- | --- |
| GER91 | Germany 91-3 | 160 | C-type lectin-like protein | ABD97357 |
| GRI90 | GRI-90 | 96 | D10L protein | CAA64081 |
| FIN00 | Finland_2000_MAN | 96 | C-type lectin domain-containing protein | ADZ29128 |
| AUS99 | Austria 1999 | 94 | C-type lectin domain-containing protein | ADZ24015 |
| GER80* | Germany_1980_EP4 | 69 | C-type lectin domain-containing protein | ADZ29555 |
| GER98* | Germany_1998_2 | 69 | C-type lectin domain-containing protein | ADZ29983 |
| FRA01* | France_2001_Nancy | 69 | C-type lectin domain-containing protein | ADZ29341 |
| NOR94* | Norway_1994_MAN | 69 | C-type lectin domain-containing protein | ADZ30411 |
| UK00* | UK2000_K2984 | 69 | C-type lectin domain-containing protein | ADZ30622 |
| GER02 | Germany_2002_MKY | 69 | C-type lectin domain-containing protein | ADZ30199 |
| GER90 | Germany_1990_2 | 69 | C-type lectin domain-containing protein | ADZ29769 |
| BR | Brighton Red | 69 | CPXV012 protein | AAM13459 |

*identical amino acid sequence
